# Supplementary material for: An evolutionarily-conserved Wnt3/β-catenin/Sp5 feedback loop restricts head organizer activity in Hydra
Source: Nat Commun. 2019 Jan 18;10:312. doi: 10.1038/s41467-018-08242-2 (PMC6338789; doi:10.1038/s41467-018-08242-2)
Supplement: Supplementary file 1 — Supplementary Information [file 41467_2018_8242_MOESM1_ESM.pdf]

An evolutionarily-conserved Wnt3/β-catenin/Sp5 feedback loop restricts head organizer activity in *Hydra*

Matthias C. Vogg[1], Leonardo Beccari[1], Laura Iglesias Ollé[1], Christine Rampon[2,3,4], Sophie Vriz[2,3,4], Chrystelle Perruchoud[1], Yvan Wenger[1], and Brigitte Galliot[1]*

Supplementary Figures **2**

Supplementary Figure 1. Upstream and coding *Sp5* sequences in *Hydra vulgaris Hm-105* strain .2
Supplementary Figure 2. Evolutionarily-conserved structure of Sp5 transcription factors 3
Supplementary Figure 3. Three Sp families, Sp1-4, Sp5 and Sp6-9, already diversified in the last common ancestor of cnidarians and bilaterians 4
Supplementary Figure 4. *HySp5* expression patterns in intact and regenerating animals..........6
Supplementary Figure 5. *HySp5* and *Wnt3* expression in *Hydra* stem cell populations 6
Supplementary Figure 6. Kinetics of *HySp5* phenotype occurrence in intact animals 7
Supplementary Figure 7. Kinetics of *HySp5* phenotype occurrence in head regenerating animals.8
Supplementary Figure 8. Knockdown of *β-catenin* delays head regeneration..........9
Supplementary Figure 9. *HySp5* phenotype occurrence requires active Wnt/β-catenin signaling.10
Supplementary Figure 10. *HySp5* antagonizes Wnt/β-catenin signaling 11
Supplementary Figure 11. Knockdown of *Sp5* in reaggregation studies 12
Supplementary Figure 12. Kinetics of *Wnt3* expression in *Sp5*(RNAi) animals 13
Supplementary Figure 13. Mapping of putative Sp5 binding sites in the *Wnt3* and *Sp5* upstream sequences in *Hydra* and teleost fish 15
Supplementary Figure 14. *HySp5* expression in Alsterpaullone-treated animals 17
Supplementary Figure 15. Interactions between Sp5 and β-Catenin or TCF1..........18
Supplementary Figure 16. Genome-wide mapping of putative Sp5 binding sites in human HEK293T cells and mouse ESCs 19
Supplementary Figure 17. Direct transcriptional targets of HySp5 and ZfSp5a. 20
Supplementary Figure 18. Overexpressing HySp5 in zebrafish embryos induces Wnt-like phenotypes..........21

Supplementary Tables.......... **22**

Supplementary Table 1. Cloning, siRNAs, qPCR and ChIP-qPCR primer sequences 22
Supplementary Table 2. DNA constructs used in this study 23

Supplementary References **24**

Supplementary Figures

```
1.......10........20........30........40........50........60........70........80........90..........
                                 -2965 ctagttctaatttagctctattacgttcgcaaagttgacaaagtcgcaaatttttttctttttca - 2901
aaagacctcccattcattttaataaagactggttctaattttgctttatcacgcttataaaaattgacaaagtcgcaagtactttgtttttttaaaagacc - 2801
tcccattcattgagataaatactagttattttcatgtatcattgaaatagtaaacaattcattctagtttttatttgtctagggcagtatttcacacctt - 2701
ccacaagtgcgaaacgttttatttacttgattgagtaaattaatttaaaataaaataaaaaaataagaacttaatgtgaaaaaaaaaacaaaacaaaaac - 2601
aataaaaaaaaaaacaatgtaaaatatttctcatagctgttgaaatattaaaaagacggaaggaaaaatataacggcaaagctaaattcttttctgcgta - 2501
ttgcttttttatctctttattgtctgtttgttgcttttcactgcattctattttgtgcttaataaatctcaatcgattttaaggaatgactaggatgtttc - 2401
attttgtatatatcaataactgaaatattaaaaatctcctcagtgcatccgttcgttagacaattggggggtattaactcaattatttcctgaatataaac - 2301
tcaacaagtaaaaaagtttcatccgtaagcaagcaagaataacgacacttgtttacatttaagaatttcttaactttatgtaaaaaacaattcttagtta - 2201
aaaacgaagtaaaagggtttttaatttttttgtttttttagttgaaaacaaattgctaaataaaacttaatttaaaaaaaaaaaaaccaaatatttaaaatgat - 2101
aaactggttaaaattaatagattatacaaaccattgtaagcatttaaaaacaattttttttttataaaaacaacaacaaaaaaaatttcacctaattactc - 2001
cgcctttgttaataaaacctccgttttacagttaaaagtaatgtatgaaccgtaatccctattacaaaaaaaatggtatattgtttataaagacgttttga - 1901
tgattgtagcttattttatattttttgttgcttttgtttgttttccttgttgttgtttttaatttgattagttatattcttacggccttacgaccacgttt - 1801
gctgttttaaatcgcgagcgttgctttgactttacacgaagcctctaaaacaaacataaagagaattcatcgaaagtaaaaaaaatcatgctgaccttcg - 1701
gtgatccgtaattgaaattgatatatatttctccctattttgacatataaatggttaaagttaatctttttattaaactcaatcaatttcaacaaaata - 1601
agtgtcaagtttactgcttatttcaagtaacaaataagtcttattgtaaataaagttattgtttaaaagggattgaaacttcaatcattattgtaataaa - 1501
gaagaatttcatgtaagatttgtatttattaaaattaaataaactaaatgaaacaaaagcgctacgtcaaatttatattttgattattaagaggaatttt - 1401
tttacctaatgtaaaactactgtaaaaatcgactgaatcaataaggtcagagagactaggtcagcgagtttggatcattaaaatcgataacaataattaa - 1301
cgatatagtttataatgataggaaacttacacttgacatttaaatgggaagtcctgaggctataacgttcgtttgtcgtgggtagataagccaattgaca - 1201
aaaccatcatcttatattttatgggcgccaaatgtttatcatgtttaatttcttttatataaatgataaaaacatttaaccacaaattattttttttat - 1101
ctccaaatgaaatcaagaacttttaagtcataaaaagtagcgacagcgccagtgataatcatagacaagtgtacacattagcttatcaaaaagtacgcta - 1001
gagtaaagcttattgttttgttttggctaggatatatccttctcgttaaaataatttgtcctacttttatatacgatattcatattttaggtttcttgt -  901
ttcgatatatatatatatttcgttatgtttgtatgtatatatgtgtttgtttgtatgtgtatgtaaaaatatgaaatatacttttttgcaaatctttgtag -  801
aagtttaataaataaagtatcaagtttaaaaattccttcgatatttttaaagcttcaatttggttgggcgtagacacattagtaattgcggtaaagatca -  701
gtaagaattctaaatagacgttaattttaaaacctggcctgccccttgattatttaatttgaaattttttaagctgtctccatttcaaccacagtatcaat -  601
gggtcggcaaaaaaagaaagattgaacgttttatcaattttaccaacgaaaaacacttaacaaatattgtagtactttttttaagtttaaatgttttttgt -  501
aaaactgttattttaaaataaacctttacttcttttttttttttttgaaatcgttttaaactgatatttaataaaagcttaaatataaatgtggtaa -  401
agttcgtaaaaccaatgaggcaggtgccggcatagatgaaagtgaaagaacaatttttttttattgaacttcacatttactgtggattgtcggaatgtttt -  301
actattaagttgatttgaagtcaaaaacaaaataacaaaatcaaccaatgaacttccttagaaattgtttaatcataaaccaatcaaatagcgttttata -  201
tcttttaaccaataagataacaattttattgttttgtcggcatcttaagatattaaaagttaatatcttttccgccttacgtattctgtttatcaccgcc -  101
tcttagaccatcccatttgtacgtaaacagagaaaatatgatcgcaacgcgccatttctcagtcagaggcgtgacattaaccccttatcaaagaagccga -    1
AGCTATTTAAGATAAGATGGCGGTTTCTTCTGATGTCTGAATGAATTGTTTCGGTTTTTTTTGAAGTAAGGATAACGTTAAGAACGATTTGAAATGCCCA    100
GCTGATTTAAATAGAATTGAAGTAGAAAGCATTCAGTTAAACTTTCTACTTTAAAGTGCAAAGACGTTCATAAGAAATACATTTCAGTAAAATTAAGTAA    200

AATGTCACCTCCAAGTCGTGTTCCAACAACAATCAGCCCAAACTTTAAAAGTCAACATCATTGTCTTAAAGAACATATTAAGTATTCACCGTTGGCATTA    300
 M  S  P  P  S  R  V  P  T  T  I  S  P  N  F  K  S  Q  H  H  C  L  K  E  H  I  K  Y  S  P  L  A  L     33

CTTGCAGCAACCTGTAAAAAAATTGGACGGCCTATCAGCCCATTAGAACAAACATCTCCTAAAAAAATTTTTCAACCATGGAATCACACGTTTGAATCAC    400
L  A  A  T  C  K  K  I  G  R  P  I  S  P  L  E  Q  T  S  P  K  K  I  F  Q  P  W  N  H  T  F  E  S  H     67

ACAATTATGACACACCTATTTCACCAAATAGCAAAGTGAGACACTTTCTAGAGACAAATTTCTCGCTTCCACCAAGTCCTCCATTAAAATCAGAGATAGT    500
 N  Y  D  T  P  I  S  P  N  S  K  V  R  H  F  L  E  T  N  F  S  L  P  P  S  P  P  L  K  S  E  I  V    100

                                                                                 Sp5-For1->
AAAAGTTCCCCCAACAATAAGACCAATGCCGATGACGAATGTAATGCAAGAAAAAGCGACTTTAAATTACTCACAAAAACTTTCTCCACCTCCGTGTCTC    600
 K  V  P  P  T  I  R  P  M  P  M  T  N  V  M  Q  E  K  A  T  L  N  Y  S  Q  K  L  S  P  P  P  C  L    133

GCATGTTCAGCTGGTCAAAAGTGCAATGGAATAAATAAAATATCTCCAGTTTTGCTTTCTCCGCCTGCCTCGCCAATCTCATGGTTATTTCCTCAAAATA    700
A  C  S  A  G  Q  K  C  N  G  I  N  K  I  S  P  V  L  L  S  P  P  A  S  P  I  S  W  L  F  P  Q  N  I    167

                                  siRNA1
TTATTCAATCTCATCCTTCTAAAGTATCAATTAACGAGCACCACATAAAAGAATATTCCGAACATTCTCAAGCTGATCCAACGCGTTTTGTAAACTACGT    800
 I  Q  S  H  P  S  K  V  S  I  N  E  H  H  I  K  E  Y  S  E  H  S  Q  A  D  P  T  R  F  V  N  Y  V    200

TTACAAAAACGTCGACTCTTCTCAAGCAAAACCTAATCTAATAATTCGACACGATAACATGATCTCCTCTACACAATCGTATAACAATCGTATATTCTCA    900
 Y  K  N  V  D  S  S  Q  A  K  P  N  L  I  I  R  H  D  N  M  I  S  S  T  Q  S  Y  N  N  R  I  F  S    233

                           siRNA2
TCTTCGCCACATTTAACTACAACATCCCACATATATTCAATGTCTACATCAATTCCTGCTCAATCGCATGCAGTTATACCGAACAGCGTTGCAACCCGAA   1000
S  S  P  H  L  T  T  T  S  H  I  Y  S  M  S  T  S  I  P  A  Q  S  H  A  V  I  P  N  S  V  A  T  R  R    267

                                                                                  siRNA3
GATGTCGTCGCTGTAAATGCCCAAACTGCATATCAGGACAACAATCTGAGCCAAATAAACCCAAGCAGCACGTATGTCATATTCCAGGGTGCGGAAAGGT   1100
 C  R  R  C  K  C  P  N  C  I  S  G  Q  Q  S  E  P  N  K  P  K  Q  H  V  C  H  I  P  G  C  G  K  V    300

        <- Sp5-Rev1
TTATGGTAAAACTAGTCACCTTAAAGCTCATTTAAGATGGCATGCTGGATTGCGTCCATTCGTTTGTAATTGGTTATTTTGCAACAAATCCTTTACTCGT   1200
 Y  G  K  T  S  H  L  K  A  H  L  R  W  H  A  G  L  R  P  F  V  C  N  W  L  F  C  N  K  S  F  T  R    333

TCTGATGAACTCCAACGTCACTTGCGAACACATACGGGCGAAAAGCGATTTGCCTGTCAAGATTGCGGCAAACGTTTTACTCGTTCCGACCATTTATCGA   1300
S  D  E  L  Q  R  H  L  R  T  H  T  G  E  K  R  F  A  C  Q  D  C  G  K  R  F  T  R  S  D  H  L  S  K    367

AACATATGAAAACACACCAAAATAAAAAACAAGAAAACACATTTGTAAAAGATACTGTCATAGAAGTGATTAAAGACAATGTCGATGAAAATTGCGATGA   1400
 H  M  K  T  H  Q  N  K  K  Q  E  N  T  F  V  K  D  T  V  I  E  V  I  K  D  N  V  D  E  N  C  D  E    400
1.......10........20........30........40........50........60........70........80........90..........

GAATGTTATGGAACTTGAAGTAAACGTTGAAAACTAAATTTAAAATTTGTTATTTATAGCTTTATAGTATATCTTCCTTTTAAATGTCTAATGTATATAA   1500
 N  V  M  E  L  E  V  N  V  E  N  *                                                                     411

CTTCAGTTTGTAAATATTGTACGTATGTAAATATTACTCCTAAATCGAAAAGTTTTTAATACTCTCTGTTTTTTAATAACGATAAAAAAATAATTAACC    1599
```

Supplementary Figure 1. Upstream and coding *Sp5* sequences in *Hydra vulgaris Hm-105* strain
Sp5 genomic sequence is written lowercase, *Sp5* transcribed sequence (c16537_g1) upper case. The deduced protein sequence is highlighted in green. DNA sequences highlighted in yellow correspond to the regions used to design siRNAs, underlined DNA stretches correspond to the primers used for subcloning and riboprobe preparation.

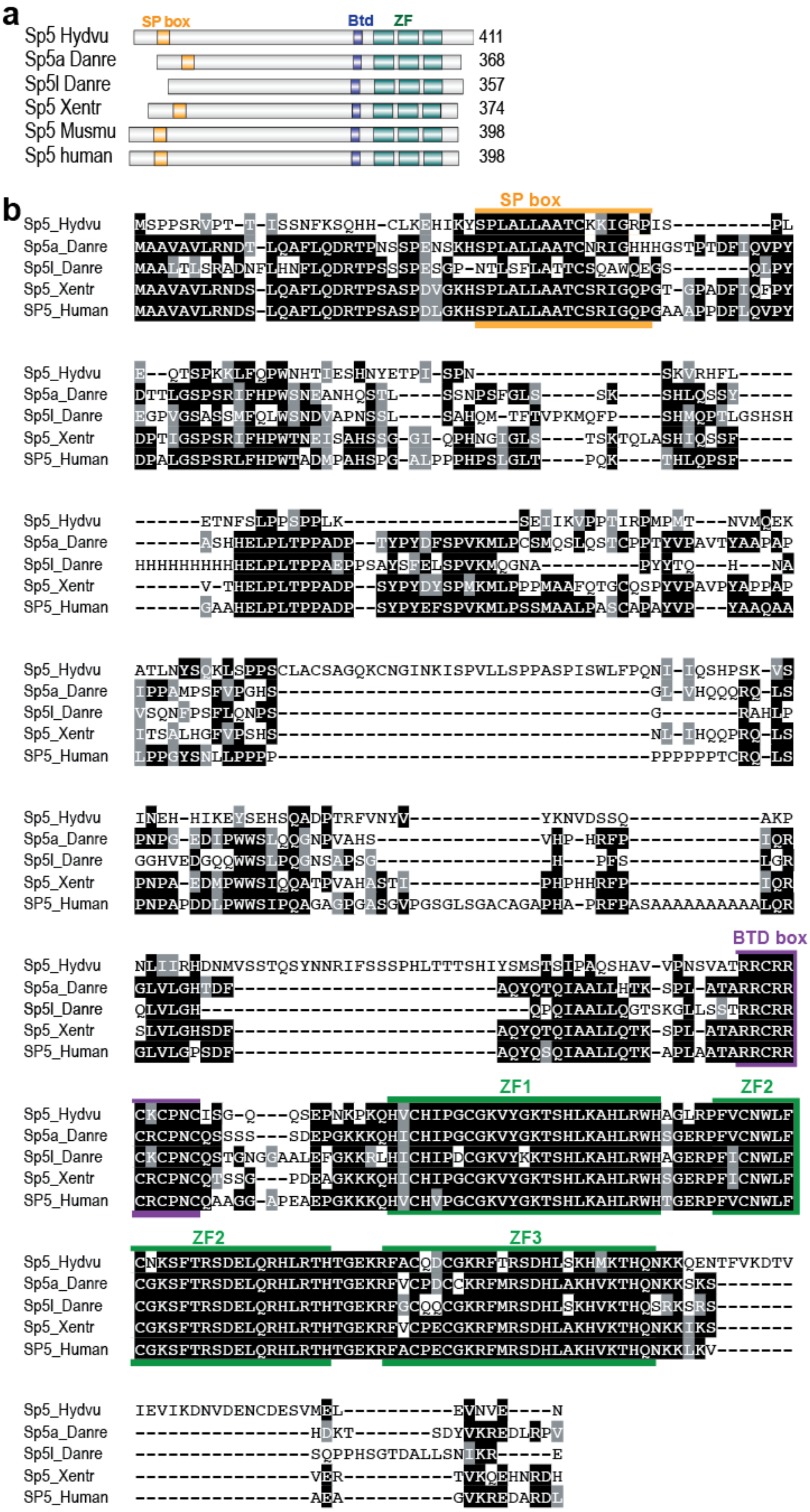

Supplementary Figure 2. Evolutionarily-conserved structure of Sp5 transcription factors

(a) Sp5 transcription factors contain a SP box (orange), a buttonhead box (Btd, purple) and a DNA-binding domain formed of three zinc finger domains (ZF, green). **(b)** Alignment of the Sp5 protein sequences from *H. vulgaris (Hydvu)*, *Danio rerio (Danre,* zebrafish), *Xenopus tropicalis* (*Xentr*) and human.

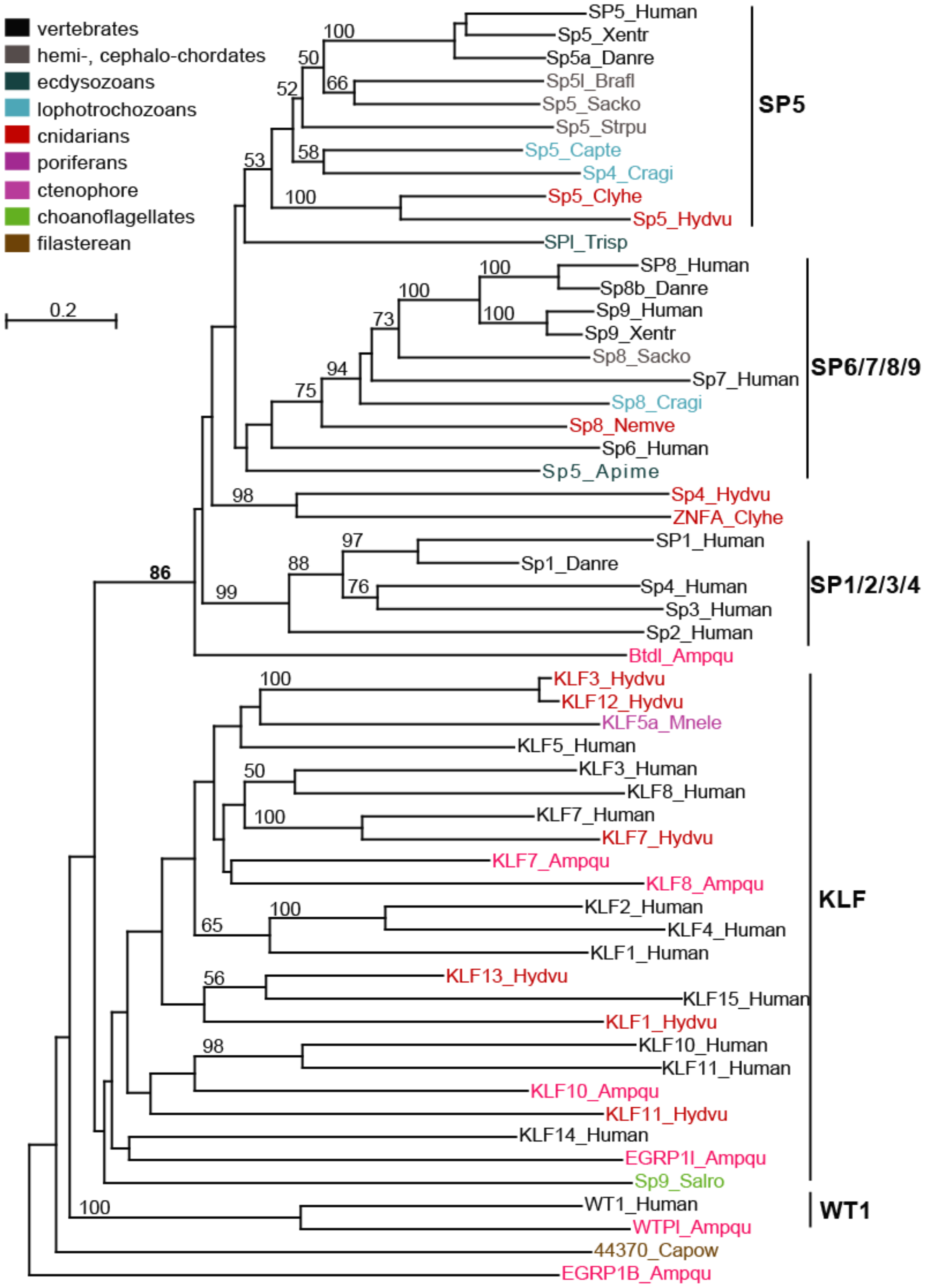

Supplementary Figure 3. Three Sp families, Sp1-4, Sp5 and Sp6-9, already diversified in the last common ancestor of cnidarians and bilaterians

The PhyML tree built on a MAFFT alignment of 56 full-length protein sequences of the Sp and KLF families tested with 100 bootstraps and rooted with two WT1 sequences. Note that a single Sp-related gene, *Btdl,* was found in the sponge *Amphimedon*, does not group with any of the three eumetazoan Sp super families. Species code: *Amphimedon queenslandica* (*Ampqu,* demosponge), *Apis mellifera (Apime*, honeybee), *Branchiostoma floridae (Brafl,* amphioxus), *Capitella telata (Capte,* annelid worm), *Capsaspora owczarzaki (Capow,* filasterean), *Clytia hemisphaerica (Clyhe,* jellyfish), *Crassostrea gigas (Cragi,* oyster), *Danio rerio (Danre,* zebrafish), *Hydra vulgaris (Hydvu)*, *Mnemiopsis leidyi (Mnele*, ctenophore), *Nematostella vectensis (Nemve,* sea anemone), *Saccoglossus kowalevskii (Sacko,* acorn worm), *Salpingoeca rosetta (Salro*, choanoflagellate), *Strongylocentrotus purpuratus (Strpu,* sea urchin), *Xenopus tropicalis* (*Xentr,* clawed frog). Note that the two major Sp families identified in bilaterians, Sp5 and Sp6-9[1] can be traced in cnidarians, whereas the unique Sp sequence identified in *Amphimedon queenslandica* cannot be affiliated to any of these, and no typical Sp sequence could be found in non-metazoan species. Therefore, the most parsimonious scenario is that a unique *Sp* gene arose at the base of metazoans to duplicate in eumetazoan ancestors.

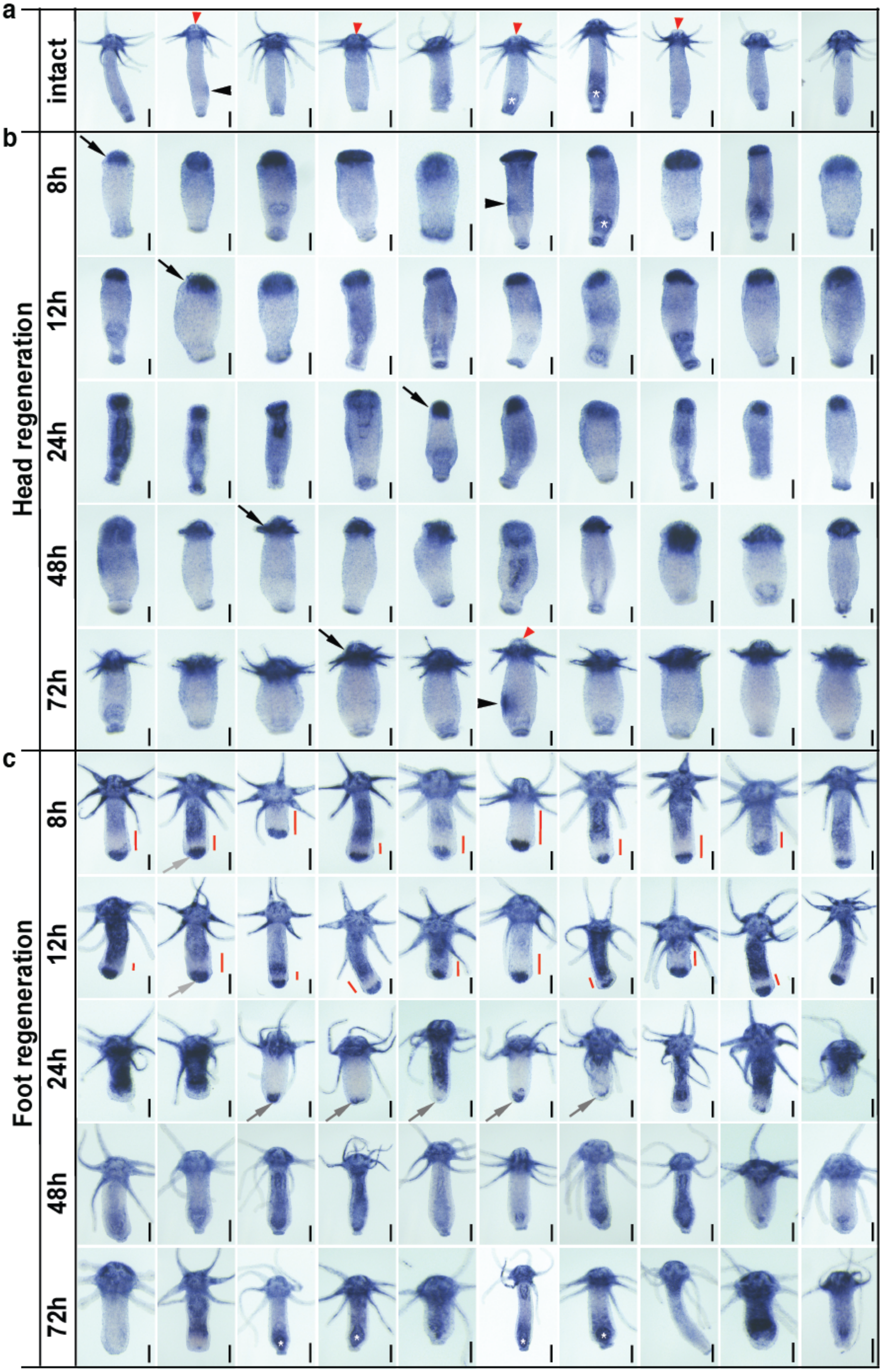
a
intact
b
Head regeneration
8h
12h
24h
48h
72h
c
Foot regeneration
8h
12h
24h
48h
72h

Supplementary Figure 4. *HySp5* expression patterns in intact and regenerating animals
(a) *HySp5* expression in intact *Hydra (Hv-Basel)* starved for 4 days is predominantly apical, although absent or strongly reduced at the most distal tip (red arrowheads). **(b, c)** *HySp5* expression in head- (b) and foot- (c) regenerating halves fixed 8, 12, 24, 48 and 72 hours after amputation (hpa, mid-gastric bisection). During head regeneration, *HySp5* expression is sustained, maximal in head-regenerating tips (black arrows), graded towards the basal end. Note the presence of a bud spot in some animals (black arrowheads). During foot regeneration, *HySp5* expression is strong at 8 and 12 hpa in foot-regenerating tips (light grey arrows) but restricted to this area as the adjacent region does not express *HySp5* (red bars); it is also transient, partially or totally lost at 24 hpa (darker grey arrows). In several animals equipped with a basal disc, the staining inside the lower part of the gastric cavity is artefactual (white asterisks). Shown are representative images of an experiment performed in duplicate. Scale bars: approximately 200 µm.

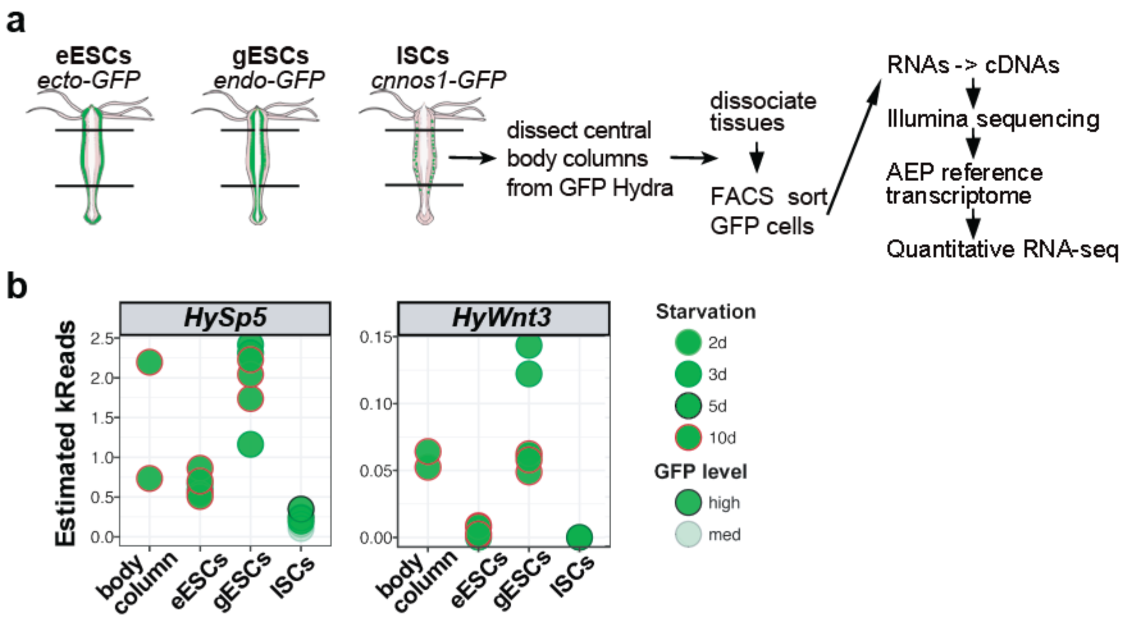

Supplementary Figure 5. *HySp5* and *Wnt3* expression in *Hydra* stem cell populations
(a) Procedure applied to perform qRNA-seq analysis of each specific stem cell population in *Hydra* (see details in [2]). We used three transgenic AEP strains that were produced and kindly provided to us by the laboratory of Thomas Bosch (Kiel). In each strain one stem cell population constitutively expresses GFP, either epidermal epithelial stem cells (eESCs, Ecto-GFP[3]), gastrodermal epithelial stem cells (gESCs, Endo-GFP[4]), or interstitial stem cells (ISCs, Cnnos1-GFP,[5]). Note that only GFP-expressing cells from the central body column were sorted by flow cytometry and analyzed for transcriptomics. **(b)** Cell type RNA-seq profiles of *HySp5* and *Wnt3*. The graphs depict the number of sequenced reads (x10^3) for *HySp5* and *HyWnt3* in the intact body column (no sorting), or in each stem cell population at different time points of starvation. Four biological replicates were tested for each condition.

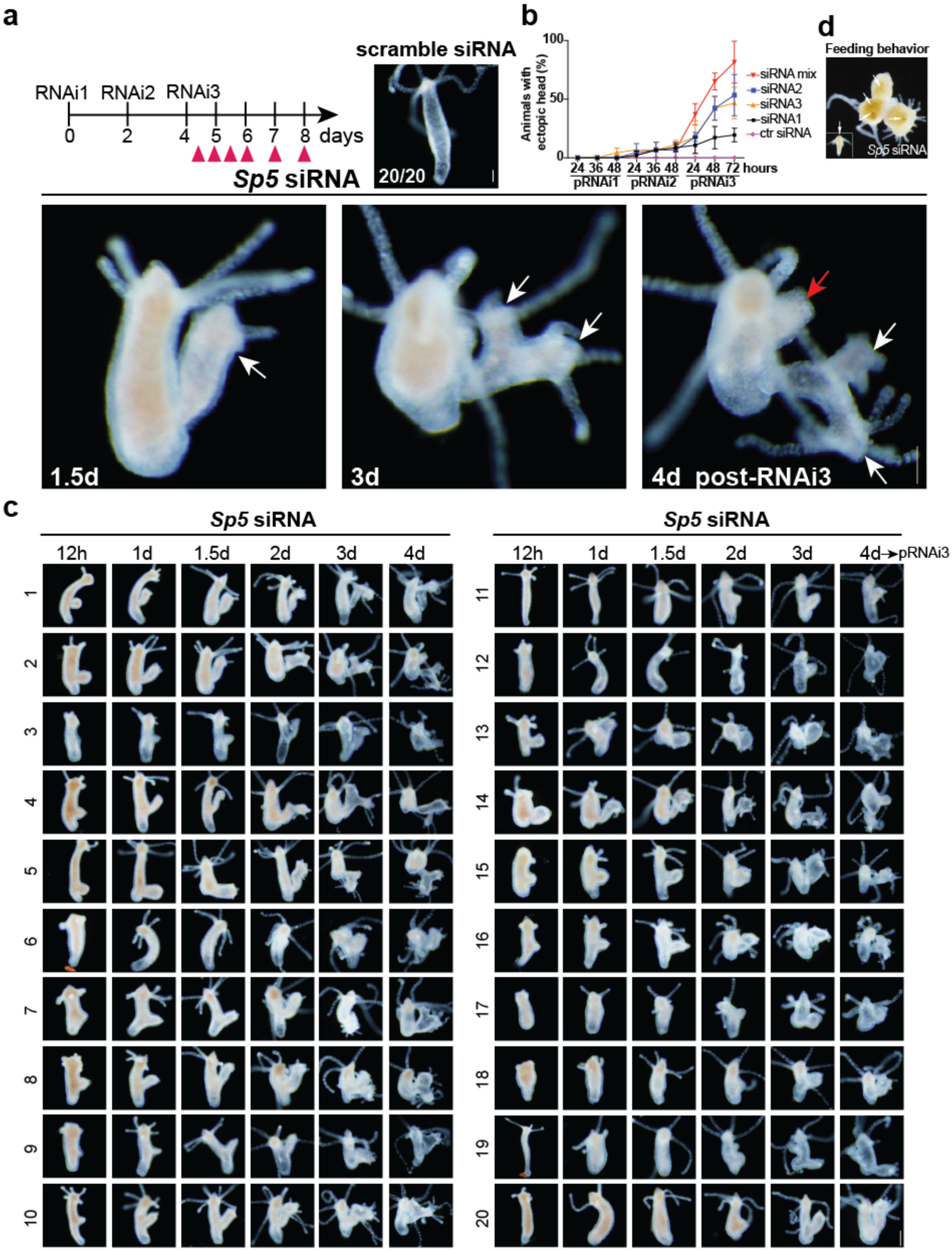

Supplementary Figure 6. Kinetics of *HySp5* phenotype occurrence in intact animals
Intact *Hydra* were electroporated three times every other day (RNAi1, RNAi2, RNAi3) either with a scramble siRNA (control) or with a mix of *HySp5* siRNAs and imaged live at various time-points after RNAi3 (red arrowheads). **(a)** Three successive views of a representative animal developing ectopic axes, first in the budding zone where they differentiate heads (white arrows), later in the upper body column where they remain headless (red arrow). **(b)** Kinetics of *Sp5* phenotype occurrence after testing *Sp5* siRNAs separately or in a mix (pool of siRNA 1-3). Note that the single siRNAs induced a multi-headed phenotype, however with a lower efficiency than the siRNA mix (3 independent experiments). Source data are provided as a Source Data file. **(c)** Multiheaded phenotype observed in 20 representative *Sp5*(RNAi) animals. Note the synchronous emergence of an ectopic axis in the budding zone of 12/20 *Sp5*(RNAi) animals already 12 hours after RNAi3, 1.5 day later in the remaining 8 animals. Ectopic axes/heads were never observed in control(RNAi) animals. **(d)** Feeding response tested in ectopic heads of *Sp5*(RNAi) animals 4 days after RNAi3. Inset: *Artemia*; white arrows: *Artemia* eyes. Shown are representative animals of an experiment performed in triplicate. Scale bars: approximately 200 μm. Error bars indicate SD.

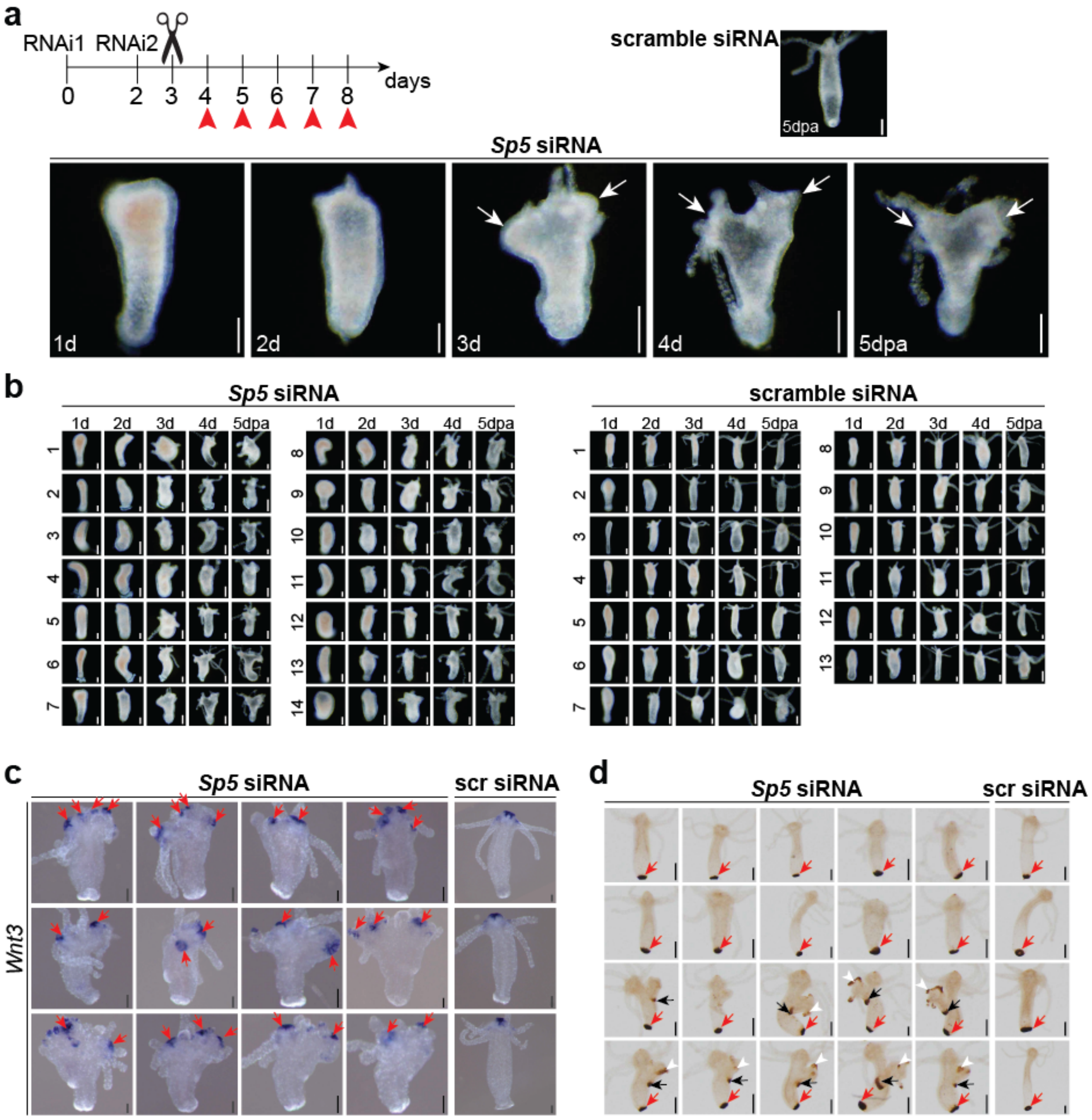

Supplementary Figure 7. Kinetics of *HySp5* phenotype occurrence in head regenerating animals

(a) Morphological changes detected in head regenerating *Sp5*(RNAi) animals after two exposures to *Sp5* siRNAs as indicated. Animals were imaged live at various time-points after mid-gastric bisection (red arrowheads). Five successive views of a representative animal regenerating multiple heads. **(b)** Multi-headed phenotype in 14 representative head regenerating *Sp5*(RNAi) animals. Note that control RNAi animals never regenerated multiple heads. **(c)** *Wnt3* expression in head regenerating *Sp5*(RNAi) animals on day 5 after mid-gastric bisection. Note the emergence of multiple *Wnt3* expressing clusters in the apex of *Sp5*(RNAi) animals (red arrows). **(d)** Detection of foot-specific peroxidase in foot regenerating *Sp5*(RNAi) animals 5 days after mid-gastric bisection. Note that foot regenerating *Sp5*(RNAi) animals never regenerated multiple heads. Red arrows: Regenerated foot; black arrows: foot of ectopic axis; white arrowheads: unspecific signal. Shown are representative animals of an experiment performed in triplicate. Scale bars: approximately 200 µm.

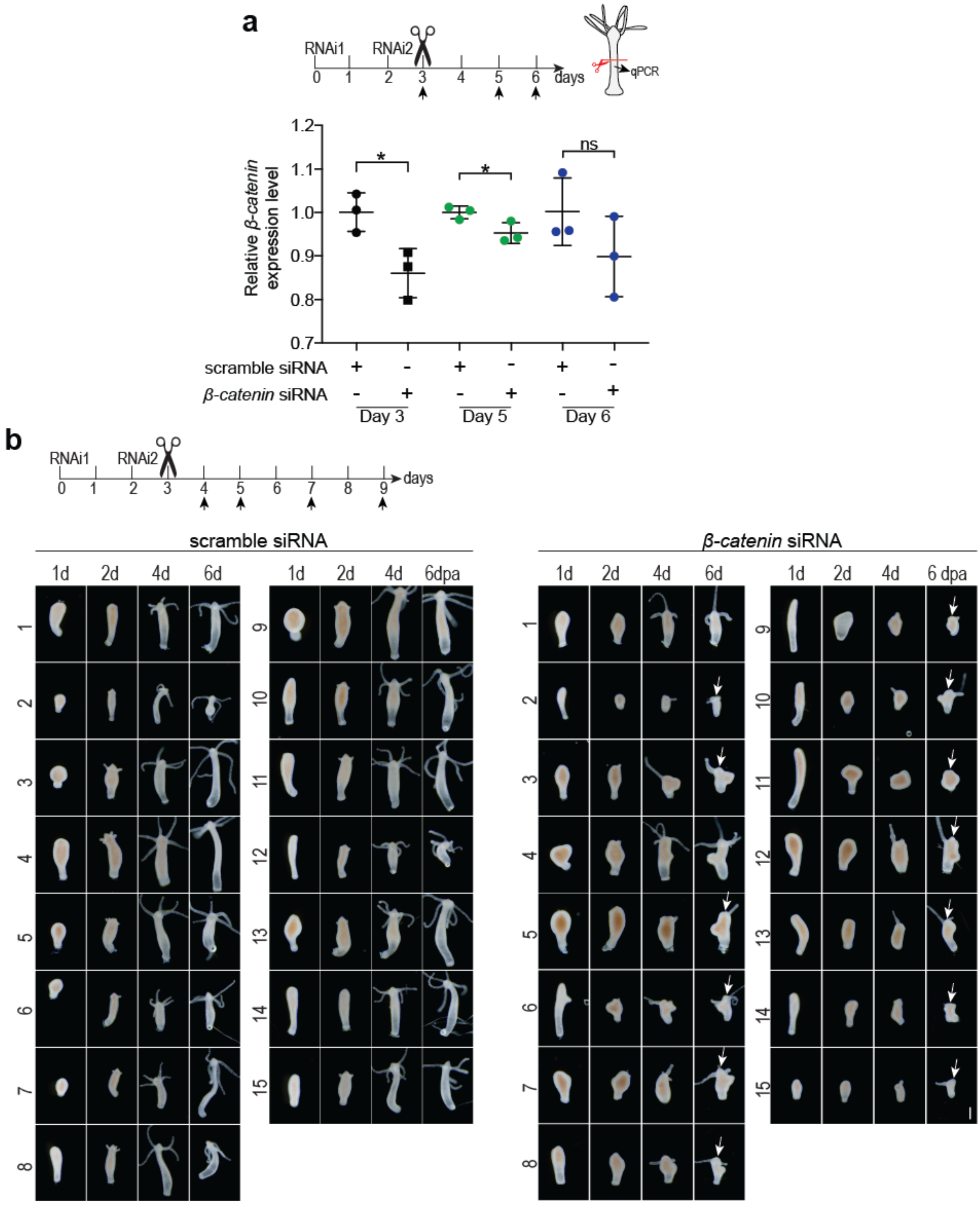

Supplementary Figure 8. Knockdown of *β-catenin* delays head regeneration

Intact *Hydra* were electroporated twice (RNAi1, RNAi2), either with a scramble siRNA or with a mix of *β-catenin* siRNAs. Head regeneration was induced by mid-gastric bisection 24 hours after RNAi2. **(a)** *β-catenin* expression detected by qPCR. Black arrows: Time points of RNA extraction. Note the significant down-regulation of *β-catenin* on day 3 and 5. Each data point represents an independent replicate. Statistical p-values: *≤ 0.05 (unpaired t test). **(b)** Scramble and *β-catenin*(RNAi) animals were imaged live at day 1, 2, 4, 6 post-amputation (black arrows). Note the delay in head regeneration after the knockdown of *β-catenin* as illustrated by a reduced number of tentacles on day 6 post-amputation (white arrows). Shown are representative animals of an experiment performed in triplicate. Scale bar: 200 µm. Error bars indicate SD.

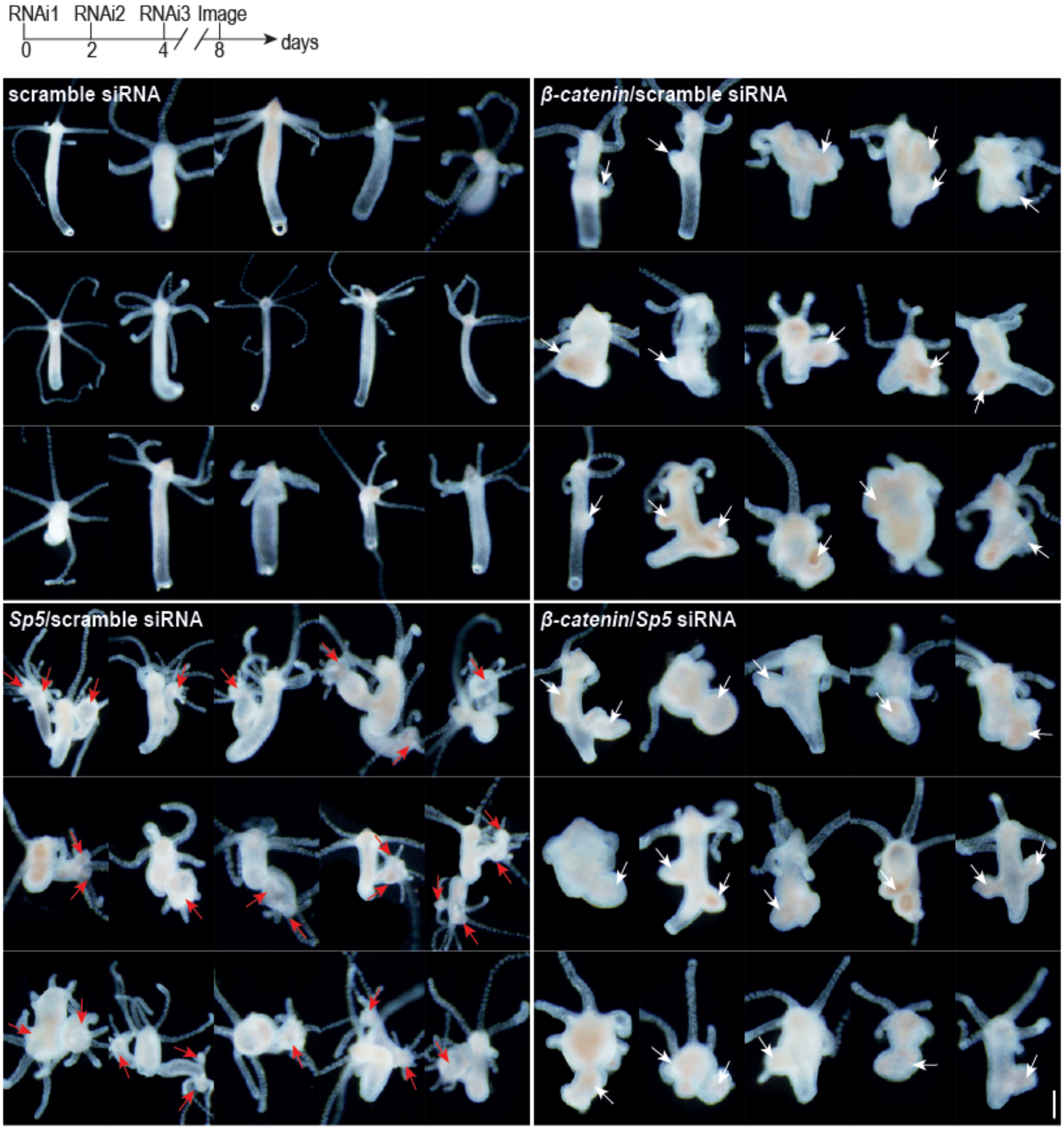

Supplementary Figure 9. *HySp5* phenotype occurrence requires active Wnt/β-catenin signaling

Intact *Hydra* were electroporated three times every other day (RNAi1, RNAi2, RNAi3) either with a scramble siRNA or a mix of *β-catenin*/scramble, *Sp5*/scramble or *β-catenin*/*Sp5* siRNAs. Shown are live animals on day 4 after RNAi3. Note that *β-catenin*/scramble RNAi animals developed ectopic bumps (white arrows) while *Sp5*/scramble RNAi animals developed ectopic heads (red arrows). Ectopic heads did no longer occur when *Sp5* was knocked-down together with *β-catenin*. Shown are representative animals of an experiment performed in duplicate. Scale bar: approximately 200 µm.

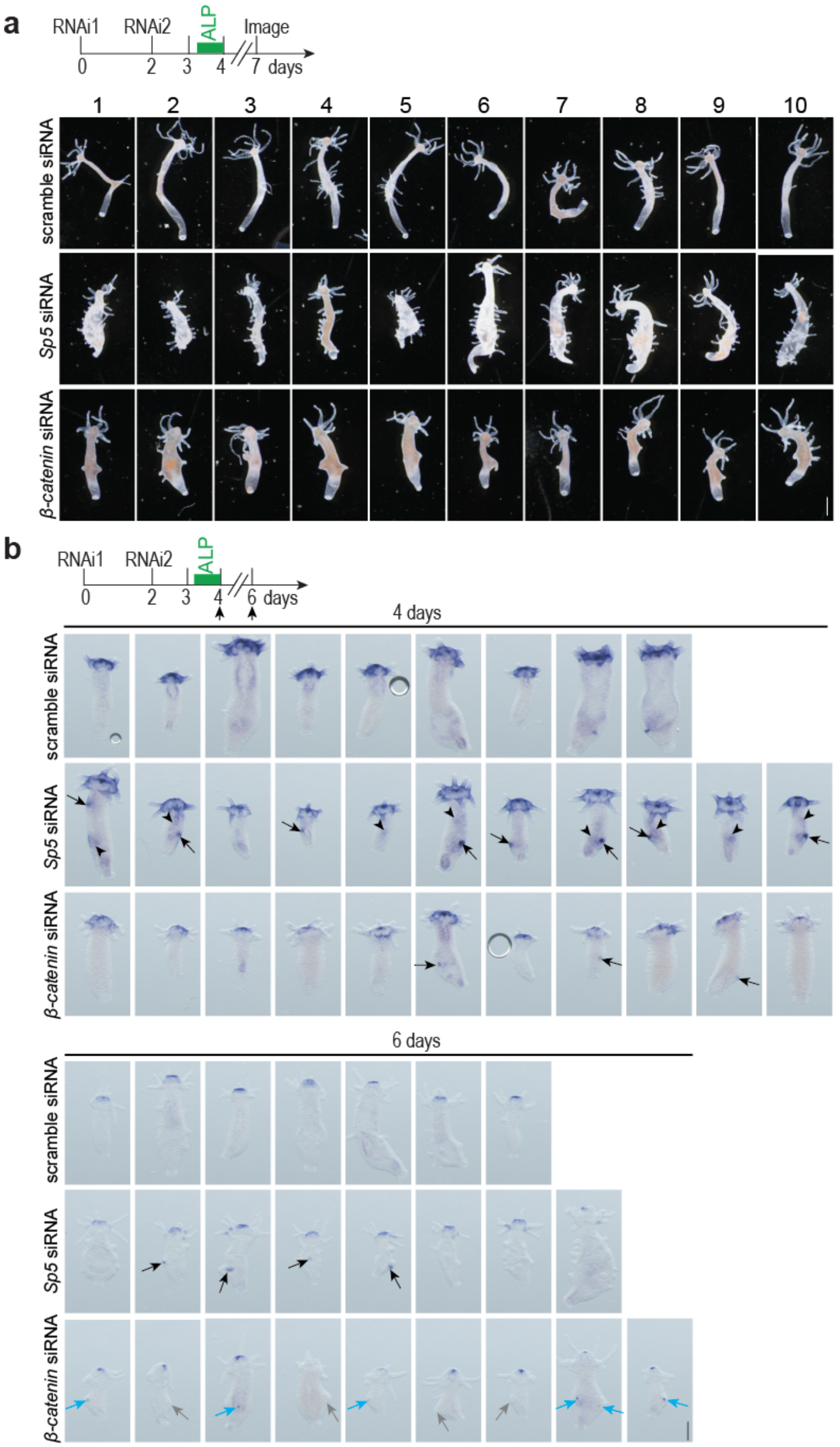

Supplementary Figure 10. *HySp5* antagonizes Wnt/β-catenin signaling

Intact *Hydra* were electroporated two times (RNAi1, RNAi2) either with a scramble, *Sp5* or *β-catenin* siRNA, followed by treatment with Alsterpaullone (ALP). **(a)** Shown are ten representative animals of an experiment performed in duplicate. Animals were fixed on day 3 after the end of the treatment with ALP. Note that the knockdown of *Sp5* enhanced ectopic tentacle formation, while the knockdown of *β-catenin* reduced ectopic tentacle formation. **(b)** Shown are representative animals fixed and detected for *Wnt3* expression either immediately (Day 4) or 2 days after the end of the treatment with ALP (Day 6). Note that the knockdown of *Sp5* increases the expression of *Wnt3* throughout the body column. Black arrows: local increase in *Wnt3* expression; black arrowheads: diffuse increase in *Wnt3*; grey arrows: *Wnt3*-negative bumps; blue arrows: *Wnt3*-positive bumps. Scale bars: approximately 200 µm.

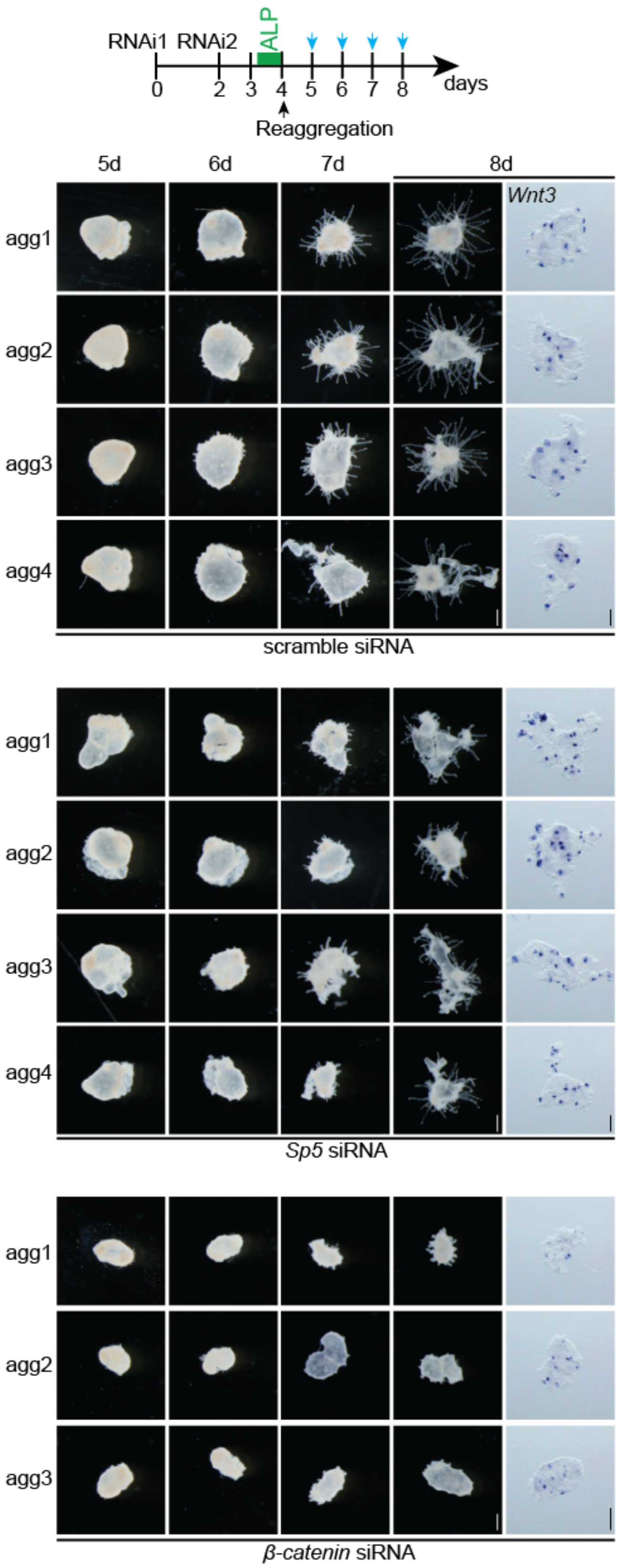

Supplementary Figure 11. Knockdown of *Sp5* in reaggregation studies

Intact *Hydra* electroporated twice (RNAi1, RNAi2) with scramble, *Sp5* or *β-catenin* siRNAs were treated with Alsterpaullone (ALP) for 18 hours and dissociated immediately after the ALP treatment to be reaggregated. Reaggregates (agg1, agg2, agg3, ...) were imaged live 1, 2, 3 or 4 days after reaggregation and then fixed four days post-dissociation (day-8) to be detected for *Wnt3* expression. Note the increased number of *Wnt3* expressing clusters in *Sp5*(RNAi) reaggregates. Shown are representative images of an experiment performed in duplicate. Scale bars: 200 µm.

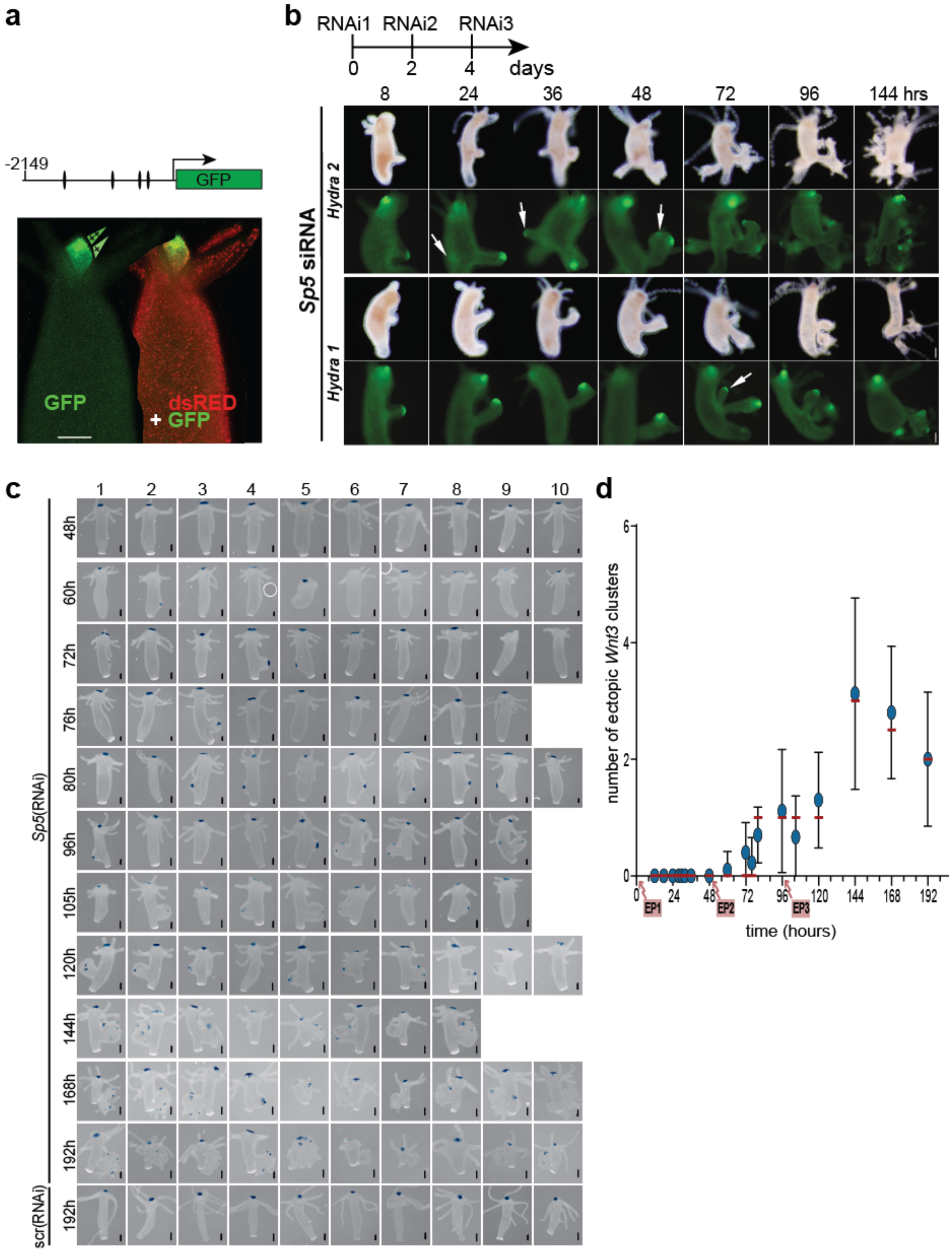

Supplementary Figure 12. Kinetics of *Wnt3* expression in *Sp5*(RNAi) animals

(a) Live transgenic *Hydra* expressing a *HyWnt3*:GFP-*HyAct*:dsRED construct where GFP expression is driven by the *Wnt3* promoter and dsRED by the ubiquitous *Hy*Actin promoter. Vertical bars: TCF binding sites. “++” and “+” indicate the maximal and intermediate GFP levels respectively. The same animal is shown in the GFP and dsRED channel. **(b)** Bright field and GFP fluorescence views of two *HyWnt3*:GFP-*HyAct*:dsRED animals (*Hydra* 1 and 2) knocked-down for *HySp5* and pictured at indicated time-points after RNAi3. Arrows: clustered GFP+ cells at the tip of ectopic axes. **(c)** Ten representative *Sp5*(RNAi) animals fixed and detected for *Wnt3* at different time points after RNAi1. Scale bars: 200 µm. **(d)** Quantification of *Wnt3*-expressing clusters for animals shown in (c). Round circles: average number of ectopic *Wnt3*-expressing clusters; red horizontal lines: median. Arrow bars indicate SD. Source data are provided as a Source Data file. (a-b) Shown are representative animals of an experiment performed in duplicate. (c) Shown are animals analyzed in one experiment.

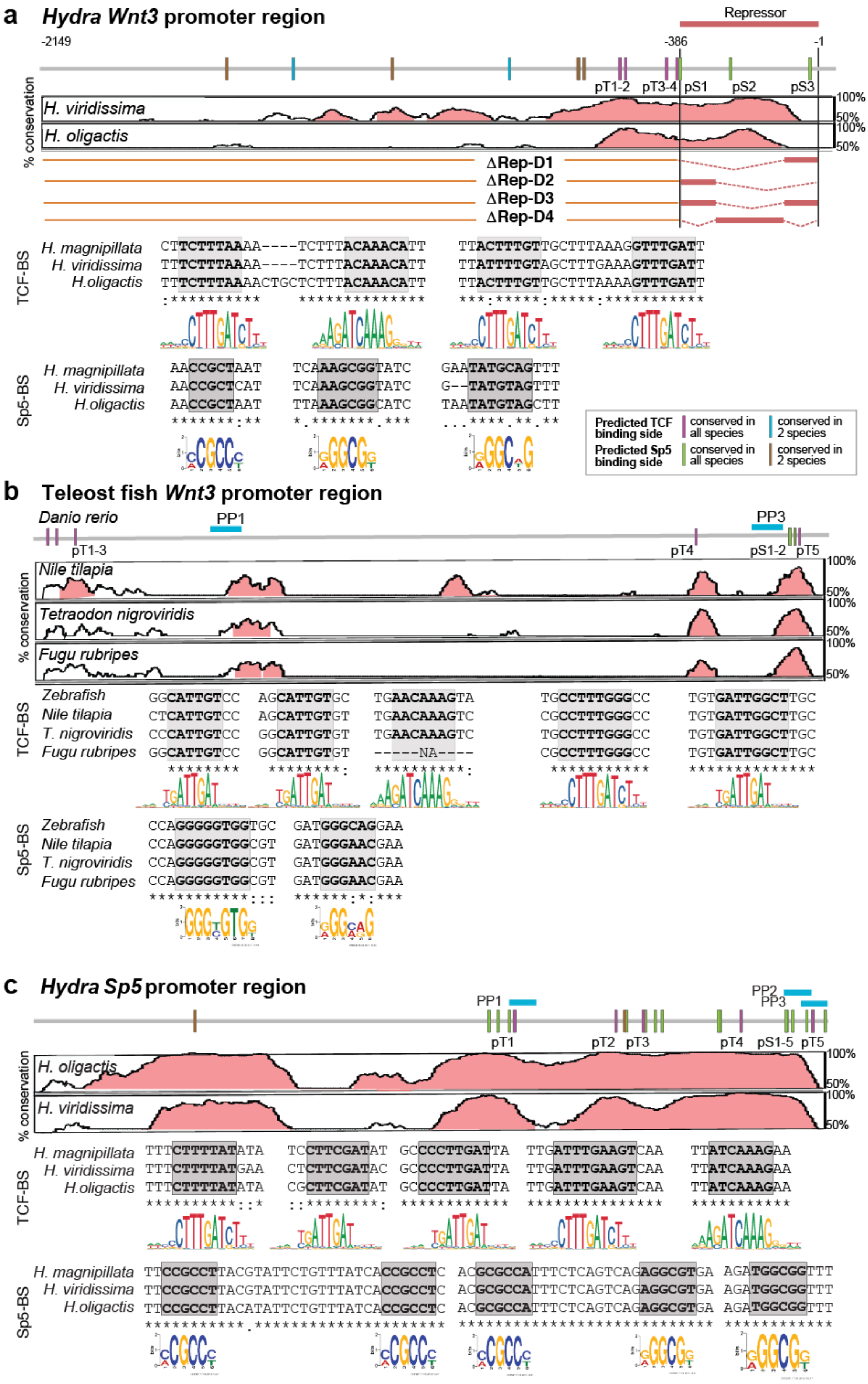

a Hydra Wnt3 promoter region
Repressor
-2149
-386
-1
pT1-2
pT3-4
pS1
pS2
pS3
% conservation
H. viridissima
H. oligactis
100%
50%
ΔRep-D1
ΔRep-D2
ΔRep-D3
ΔRep-D4
TCF-BS
H. magnipillata
H. viridissima
H.oligactis
CTTCTTTAAAA----TCTTTACAAACATT
TTTCTTTAAAA----TCTTTACAAACATT
TTTCTTTAAAACTGCTCTTTACAAACATT
TTACTTTGTTGCTTTAAAGGTTTGATT
TTATTTTGTAGCTTTGAAAGTTTGATT
TTACTTTGTTGCTTTAAAAGTTTGATT
Sp5-BS
AACCGCTAAT
AACCGCTCAT
AACCGCTAAT
TCAAAGCGGTATC
TCAAAGCGGTATC
TTAAAGCGGCATC
GAATATGCAGTTT
G--TATGTAGTTT
TAATATGTAGCTT
Predicted TCF binding side
Predicted Sp5 binding side
conserved in all species
conserved in 2 species
b Teleost fish Wnt3 promoter region
Danio rerio
PP1
PP3
pT1-3
pT4
pS1-2
pT5
Nile tilapia
Tetraodon nigroviridis
Fugu rubripes
Zebrafish
T. nigroviridis
GGCATTGTCC
CTCATTGTCC
CCCATTGTCC
GGCATTGTCC
AGCATTGTGC
AGCATTGTGT
GGCATTGTGT
GGCATTGTGT
TGAACAAAGTA
TGAACAAAGTC
TGAACAAAGTC
-----NA----
TGCCTTTGGGCC
CGCCTTTGGGCC
TGCCTTTGGGCC
CGCCTTTGGGCC
TGTGATTGGCTTGC
TGTGATTGGCTTGC
TGTGATTGGCTTGC
TGTGATTGGCTTGC
CCAGGGGGTGGTGC
CCAGGGGGTGGCGT
CCAGGGGGTGGCGT
CCAGGGGGTGGCGT
GATGGGCAGGAA
GATGGGAACGAA
GATGGGAACGAA
GATGGGAACGAA
c Hydra Sp5 promoter region
PP1
PP2
PP3
pT1
pT2
pT3
pT4
pS1-5
pT5
TTTCTTTTATATA
TTTCTTTTATGAA
TTTCTTTTATATA
TCCTTCGATAT
CTCTTCGATAC
CGCTTCGATAT
GCCCCTTGATTA
GCCCCTTGATTA
GCCCCTTGATTA
TTGATTTGAAGTCAA
TTGATTTGAAGTCAA
TTGATTTGAAGTCAA
TTATCAAAGAA
TTATCAAAGAA
TTATCAAAGAA
TTCCGCCTTACGTATTCTGTTTATCACCGCCTC
TTCCGCCTTACGTATTCTGTTTATCACCGCCTC
TTCCGCCTTACATATTCTGTTTATCACCGCCTC
ACGCGCCATTTCTCAGTCAGAGGCGTGA
ACGCGCCATTTCTCAGTCAGAGGCGTGA
ACGCGCCATTTCTCAGTCAGAGGCGTGA
AGATGGCGGTTT
AGATGGCGGTTT
AGATGGCGGTTT

Supplementary Figure 13. Mapping of putative Sp5 binding sites in the *Wnt3* and *Sp5* upstream sequences in *Hydra* and teleost fish

Map showing the location of the putative Sp5 (pS) and TCF (pT) binding sites along the upstream sequences of *Hydra Wnt3* **(a)**, zebrafish *Wnt3* **(b)** and *Hydra Sp5* **(c)**. Sp5 binding sites were identified using the FIMO tool (see material and methods) and the consensus matrixes identified from the analysis of the HySp5 and ZfSp5a ChIP-seq dataset using MEME ChIP suite (see Methods). TCF binding sites (TCF-BS) were identified using the TCF1-LEF1 and TCF4 consensus matrixes available from the MultiTF tool implemented in Ecr browser. The evolutionary conservation of the putative Sp5 binding sites (Sp5-BS) was determined by comparing their sequence across three *Hydra* or four teleost fish species using the Vista alignment tool. Green bars: Sp5-BS conserved in all analyzed species, brown bars: Sp5-BS conserved in only two species, magenta bars: TCF-BS conserved in all analyzed species, blue bars : TCF-BS conserved in only two species ; Blue boxes: PP (primer pairs) regions tested in ChIP-qPCR experiments. A multispecies alignment of the sequences corresponding to each predicted TCF-BS (upper row) or Sp5-BS (lower row) is shown below the Vista plot (pink peaks) with the corresponding consensus matrix. The predicted BS sequences are written bold on a gray background. Stars mark nucleotides identical in all species, semi-columns the nucleotides conserved in 2/3 or 3/4 species. When the putative BS is located in the negative strand the reverse complement version of the corresponding matrix is shown. In the case of the *HySp5* promoter only the putative Sp5-BS located within the regions enriched in the ChIP-qPCR analysis (PP1, PP2, PP3) are shown. In (a) the *Wnt3* promoter sequences tested in deletion reporter constructs are schematized.

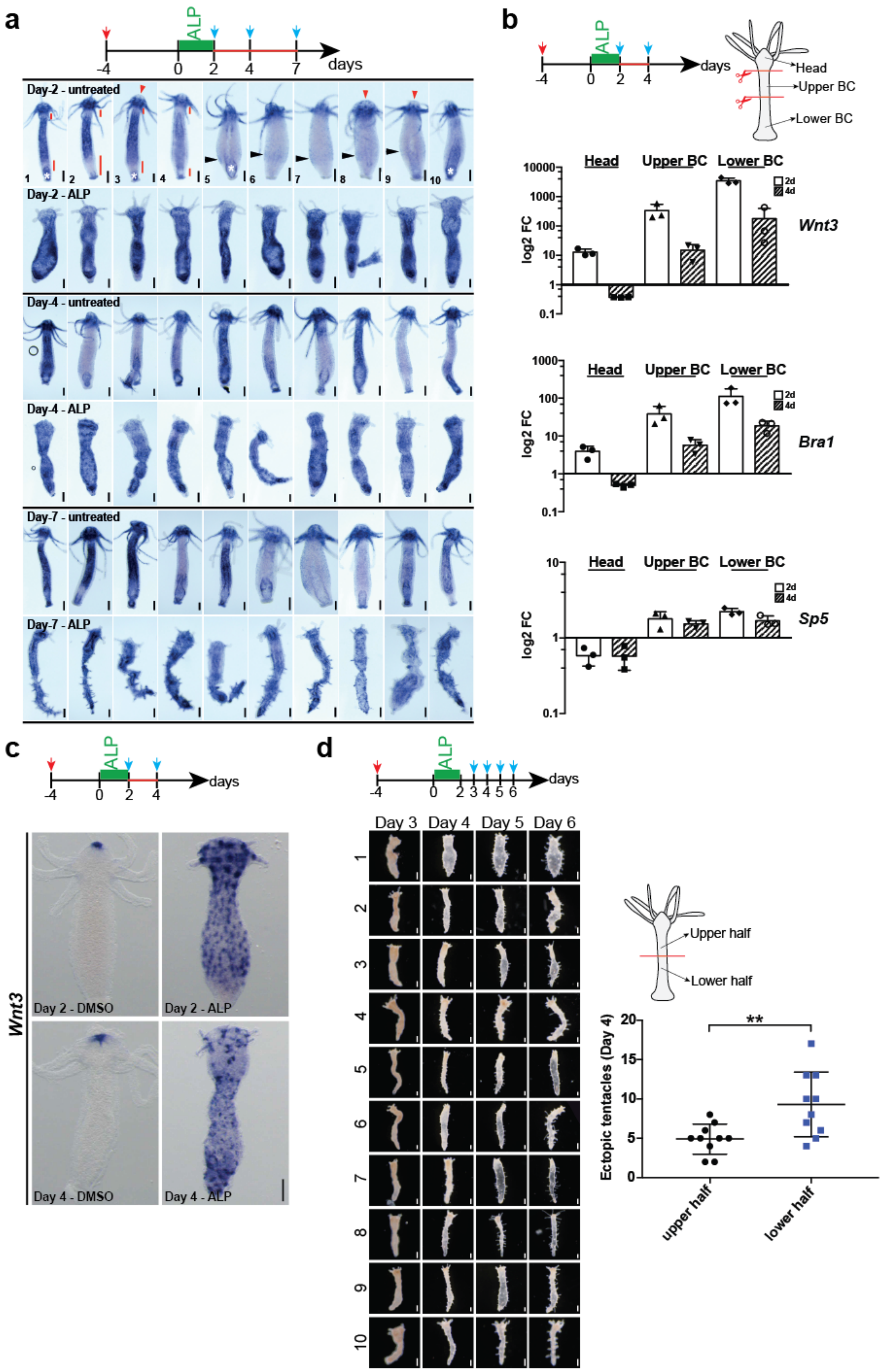

a
ALP
-4
0
2
4
7
days
Day-2 - untreated
Day-2 - ALP
Day-4 - untreated
Day-4 - ALP
Day-7 - untreated
Day-7 - ALP
b
ALP
-4
0
2
4
days
Head
Upper BC
Lower BC
10000
1000
100
10
1
0.1
log2 FC
2d
4d
Wnt3
Bra1
Sp5
c
ALP
days
Wnt3
Day 2 - DMSO
Day 2 - ALP
Day 4 - DMSO
Day 4 - ALP
d
ALP
-4
0
2
3
4
5
6
days
Day 3
Day 4
Day 5
Day 6
Upper half
Lower half
Ectopic tentacles (Day 4)
20
15
10
5
0
**
upper half
lower half

Supplementary Figure 14. *HySp5* expression in Alsterpaullone-treated animals

(a) *HySp5* expression in intact animals exposed for two days to Alsterpaullone (ALP) and fixed either immediately (Day 2), or transferred to HM and fixed two and five days later (Day 4, Day 7). Red arrow: last feeding, blue arrows: fixation days. Note that in all animals *HySp5* expression is high in the head region but low at the very apical tip (red arrowheads). In animals 1-4 (Day 2 untreated animals) *HySp5* expression is high in the body column with two adjacent regions where *HySp5* expression is low (red bars); in animals 5-10, *HySp5* expression is low in the body column and several show a higher level of *HySp5* expression in the budding zone (black arrowheads). Upon ALP treatment, *HySp5* is increased in the body column of all animals, and ectopic tentacles form dots of enhanced *HySp5* expression at Day 4, and become visible at Day 7. Shown are representative animals of an experiment performed in triplicate. **(b)** Treatment of intact *Hydra* with ALP for 2 days followed by detection of *Wnt3*, *Bra1* and *Sp5* expression by qPCR in head as well as upper and lower body column tissue. Blue arrows: Days of RNA extraction. Note the up-regulation of *Wnt3*, *Bra1* and *Sp5* in body column tissue on Day 2 and Day 4, the up-regulation of *Wnt3* and *Bra1* in head tissue on Day 2 and down-regulation on Day 4 as well as the down-regulation of *Sp5* in head tissue at both time points. Each point represents an independent replicate. **(c)** Treatment of *Hydra* with ALP for 2 days and detection of *Wnt3* on Day 2 and Day 4 (blue arrows). Note the reduction of *Wnt3* expression on Day 4 compared to Day 2 in head and body column tissue. Shown are representative animals of an experiment performed in triplicate. **(d)** Intact *Hydra* were treated with ALP for 2 days and imaged on Day 1-4 (blue arrows) after the end of the ALP treatment (left panel). Shown are representative animals of an experiment performed in triplicate. Quantification of ectopic tentacles in the upper and lower body column two days after the end of the ALP treatment (Day 4) (right panel). Note that ectopic tentacles first occurred in the lower half, which is consistent with a higher expression of *Wnt3* in lower than upper body column tissue (see panel b). Each data point represents one animal. Statistical p-values: $^{**}\leq 0.01$ (unpaired t test). Scale bars: approximately 200 μm. Arrow bars indicate SD.

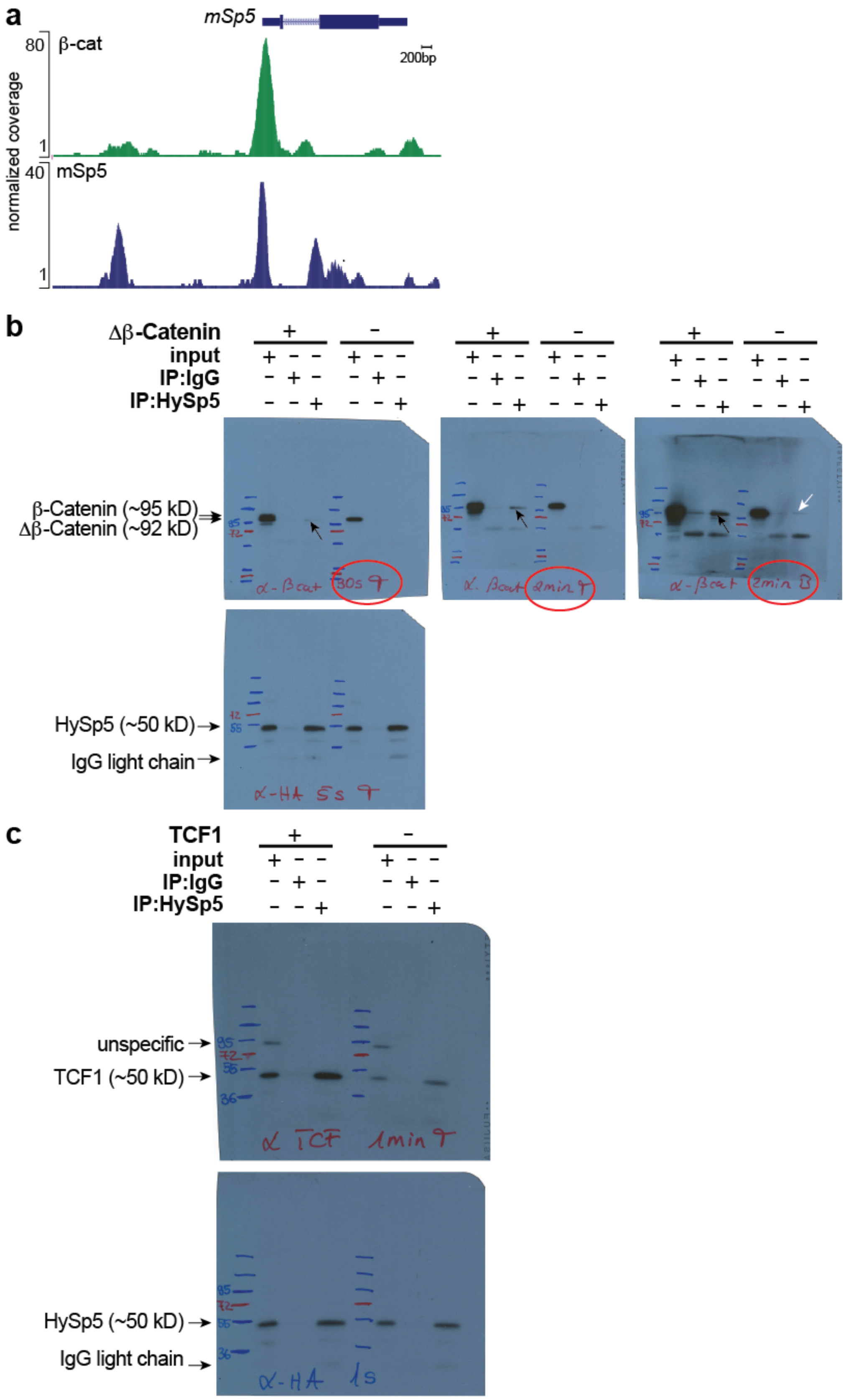

Supplementary Figure 15. Interactions between Sp5 and β-Catenin or TCF1

(a) ChIP-seq profile showing the genomic occupancies of the mouse Sp5 and β-catenin over the genomic region encompassing the *Sp5* locus in mouse ES cells. The profiles were obtained by re-mapping publicly available datasets[6,7]. Note the overlap in the occupancies of Sp5 and β-catenin in the vicinity of the *Sp5* transcriptional start site. **(b-c)** Nuclear extracts were prepared from HEK293T cells transiently expressing HySp5_HA protein or not, in

the presence of a constitutively active form of human β-Catenin (huΔβ-Cat) (b) or human TCF1 (c). **(b)** HySp5 and β-Catenin interaction after anti-HA immunoprecipitation, Western blotting and immunodetection with the anti-β-Catenin antibody (upper, at two distinct exposure times), or the anti-HA antibody (lower). Note that HySp5 interacts with exogenous (black arrows) and endogenous (white arrow) β-Catenin. T: Top; B= Bottom. **(c)** HySp5 and TCF1 interaction detected after anti-HA immunoprecipitation, Western blotting and immunodetection with the anti-TCF1 antibody. Note that HySp5 interacts with exogenous and endogenous TCF1. All Co-IP experiments were performed twice with extracts prepared independently.

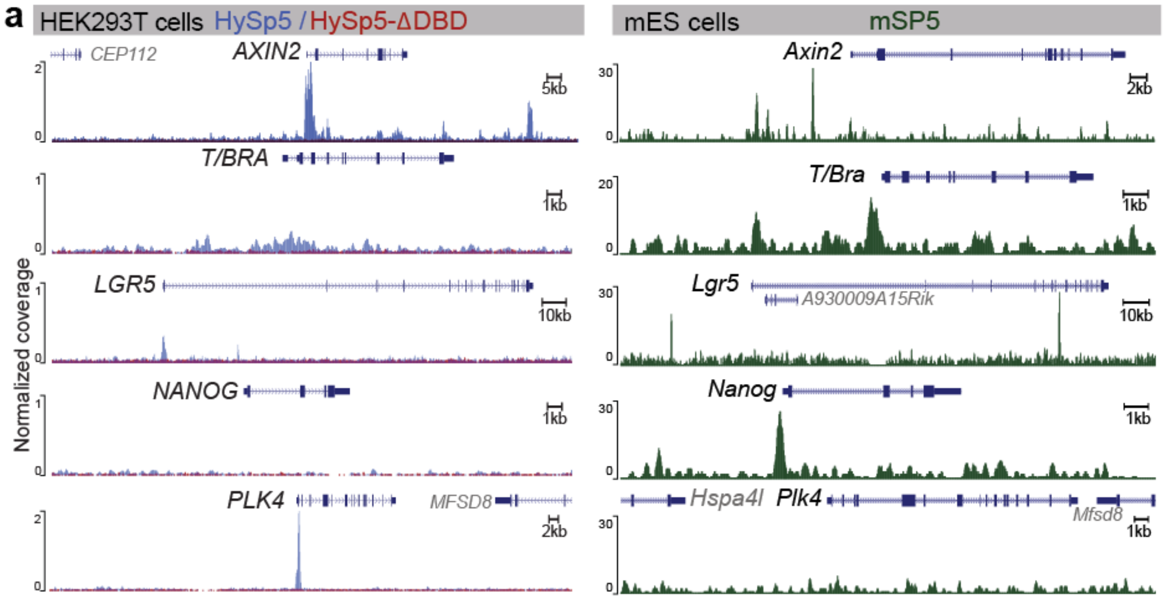

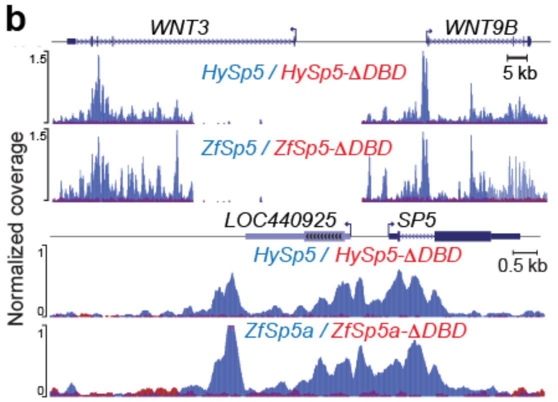

c

GOterm Description	FDR	Enrich.
Wnt signaling pathway	1.2e-16	1.89
Cell surface receptor signaling pathway involved in cell-cell signaling	1.4e-16	1.88
Plasma membrane bounded cell projection morphogenesis	1.3e-11	1.88
Cell projection morphogenesis	1.9e-11	1.87
Regionalisation	1.9e-12	1.85
Regulation of cell morphogenesis involved in differentiation	2.7e-14	1.84
Neuron projection morphogenesis	2.6e-10	1.83
Negative regulation of transcription by RNA polymerase II	7.5e-41	1.82
Tube morphogenesis	6.0e-13	1.82
Ras protein signal transduction	3.2e-11	1.80

Supplementary Figure 16. Genome-wide mapping of putative Sp5 binding sites in human HEK293T cells and mouse ESCs

(a) ChIP-seq analysis showing the binding profiles of HySp5 expressed in HEK293T cells (left panels) within the genomic regions of known Wnt target genes, compared to the previously reported genomic occupancies of the mouse Sp5 in the corresponding mouse ortholog loci[7] (right panels). The control recombinant protein HySp5-ΔDBD does not show any significant enrichment over the same genomic regions. **(b)** ChIP-seq analysis showing the genomic occupancies of the HySp5 and ZfSp5a proteins (blue) in the genome of HEK293T cells expressing these proteins. No enrichment is scored when HEK293T cells express Sp5 proteins lacking the DBD (red). **(c)** Table summarizing the 10 most enriched GO terms associated with the genes assigned to the Sp5-enriched regions in HEK293T cells expressing HySp5 and ZfSp5a. GO term search was performed using the Gorilla software to compare the genes assigned to Sp5 bound regions in both HySp5 and ZfSp5a ChIP-seq experiments versus the full list of human genes.

a

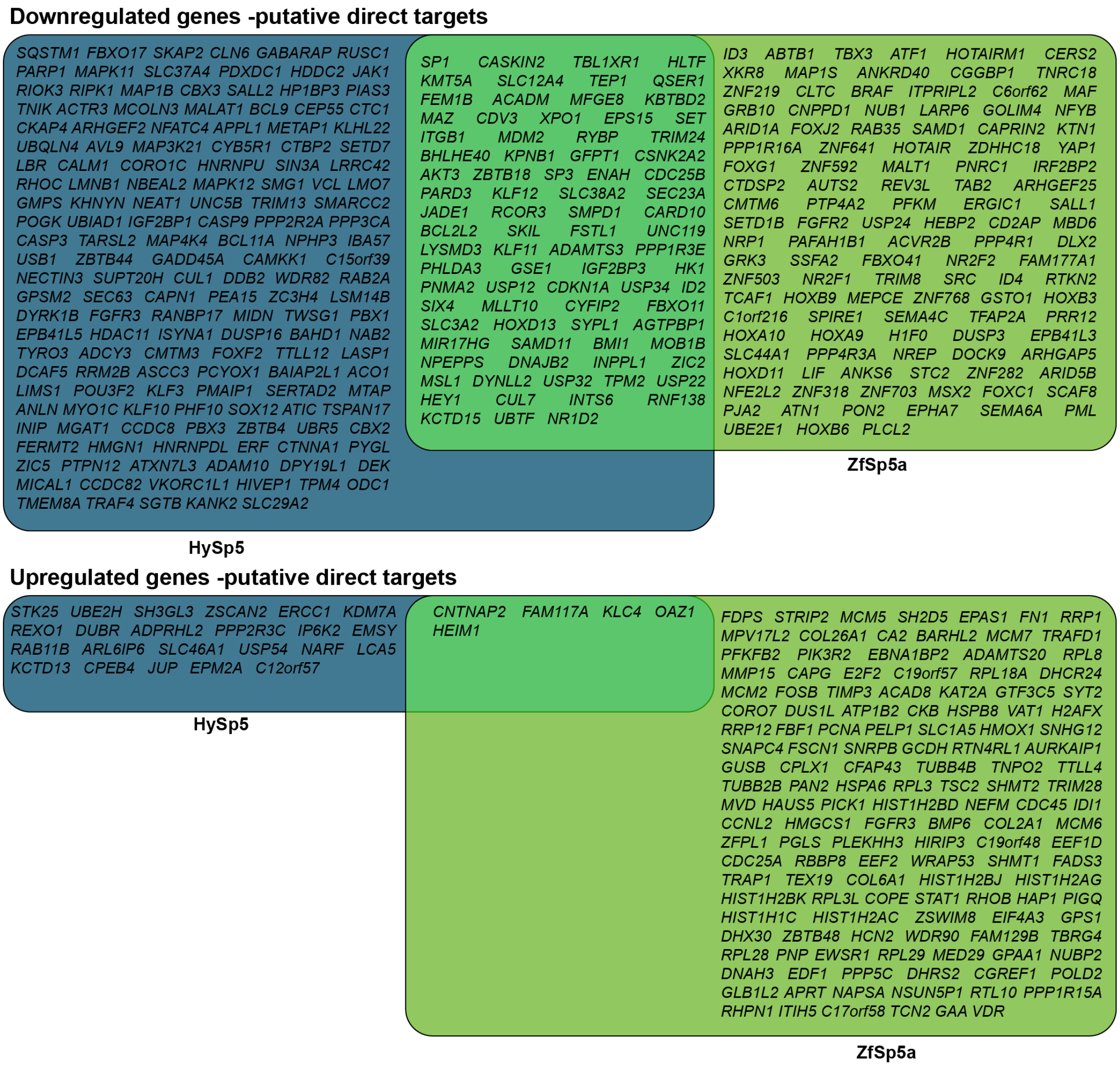

b

	GO term description	FDR
ZfSp5a downreg. targets	Positive regulation of cellular process	3.4E-04
	Negative regulation of transcription, DNA-templated	5.9E-04
	Regulation of metabolic process	1.7E-03
	Positive regulation of biological process	2.3E-03
	Cellular macromolecule metabolic process	2.9E-03
HySp5 downreg. targets	Cellular response to stimulus	1.1E-04
	Regulation of cellular process	1.6E-04
	Cellular response to stress	2.5E-04
	Regulation of cellular metabolic process	2.6E-04
	Positive regulation of macromolecule metabolic process	6.1E-04
HySp5-ZfSp5a downreg. targets	Regulation of transcription, DNA-templated	9.5E-08
	Negative regulation of transcription, DNA-templated	1.0E-07
	Regulation of transcription by RNA polymerase II	7.1E-07
	Regulation of metabolic process	8.4E-07
	Anatomical structure morphogenesis	8.7E-07

	GO term description	FDR
ZfSp5a upreg. targets	Organic cyclic compound metabolic process	2.5E-04
	Nucleobase-containing compound metabolic process	6.6E-04
	Heterocycle metabolic process	7.7E-04
	Cellular aromatic compound metabolic process	7.9E-04
	Cellular nitrogen compound metabolic process	2.0E-03

Supplementary Figure 17. Direct transcriptional targets of HySp5 and ZfSp5a.
(a) Venn diagram showing the genes repressed (Top) or activated (Bottom) upon HySp5 or ZfSp5a overexpression in HEK293T cells and associated with HySp5 or ZfSp5a bound elements. **(b)** Summary of the 5 most significantly enriched GO term categories (based on their FDR value) for the different subset of genes represented in the Venn diagram in (a). No significatly enriched GO term categories were identifed for the HySp5 specific and HySp5-ZfSp5a common upregulated targets. When more than 10 significantly enriched GO term categories were identified (FDR<0,05) the REVIGO tool was used to group related GO term classes using 0.7 as treshold for allowed similarity.

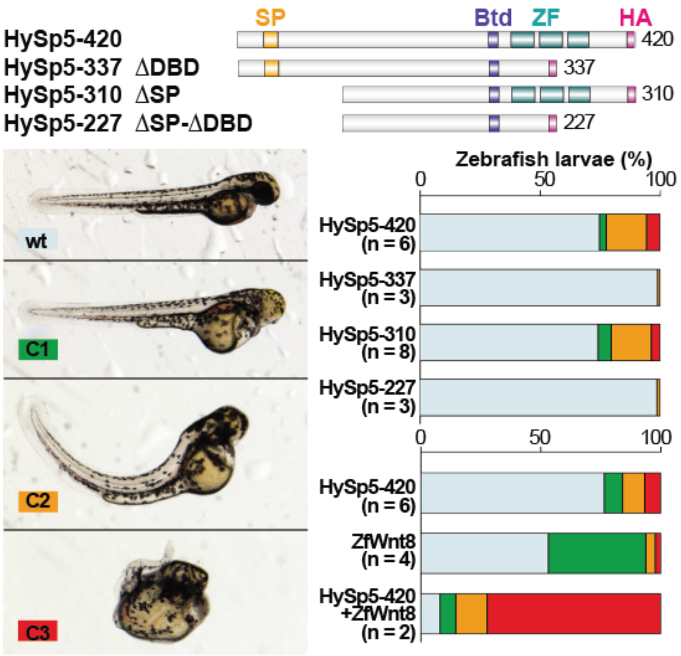

mRNA	[conc] pg	N	†% (n)	Sn	wt % (n)	class1 % (n)	class2 % (n)	class3 % (n)	Total % of abnormal embryos
**HySp5-420**	400	79	10.1% (8)	71	**74.7%** (53)	**2.8%** (2)	**16.9%** (12)	**5.6%** (4)	**25.3%**
**HySp5-420**	400	128	7.0% (9)	119	**76.5%** (91)	**7.6%** (9)	**9.2%** (11)	**6.7%** (8)	**23.5%**
**HySp5-337 ΔDBD**	400	96	2.1% (2)	94	**98.9%** (93)	**0.0%** (0)	**1.1%** (1)	**0.0%** (0)	**1.1%**
**HySp5-310 ΔSP**	400	56	3.6% (2)	54	**74.1%** (40)	**5.5%** (3)	**16.7%** (9)	**3.7%** (2)	**25.9%**
**HySp5-227 ΔSP-ΔDBD**	400	74	4.1% (3)	71	**98.6%** (70)	**0.0%** (0)	**1.4%** (1)	**0.0%** (0)	**1.4%**
**HySp5-420**	400	128	7.0% (9)	119	**76.5%** (91)	**7.6%** (9)	**9.2%** (11)	**6.7%** (8)	**23.5%**
**ZfWnt8**	4	138	7.3% (10)	128	**53.1%** (68)	**40.6%** (52)	**3.9%** (5)	**2.3%** (3)	**46.9%**
**HySp5-420 + ZfWnt8**	400+4	155	11.6% (18)	137	**8.0%** (11)	**6.6%** (9)	**13.1%** (18)	**72.3%** (99)	**82%**

Supplementary Figure 18. Overexpressing HySp5 in zebrafish embryos induces Wnt-like phenotypes

To test whether HySp5 is a mediator of the Wnt pathway, we injected *HySp5* mRNAs, either full-length (HySp5-FL) or lacking the SP box (HySp5-ΔSP) into zebrafish embryos and looked at larval morphology on day-2 post-fertilization (dpf). **(upper panel)** Wnt-like phenotypes detected in 2 days old zebrafish larvae overexpressing HySp5 (HySp5-420). These phenotypes were scored in three classes: no eyes (C1); no eyes + curly axis (C2); no eyes, underdeveloped axis and curly tail (C3). The HySp5 constructs lacking the DNA-binding domain do not affect embryonic development, whereas co-injecting ZfWnt8 with HySp5-420 increases the phenotypic penetrance. The number of independent experiments (n) is indicated for each construct and the graphs show one representative experiment. **(lower panel)** Table showing the scoring of the embryonic phenotypes identified in zebrafish overexpressing HySp5 or ZfWnt8. One representative experiment is shown. All embryos were produced from wild-type parental strains and analyzed at 48 hpf. N = number of injected embryos; †%(n) = percentage (number) of dead embryos; Sn = number of surviving embryos. Given the similarities with the morphological defects obtained when the zebrafish β-catenin or Wnt8 are overexpressed during development[8,9], we deduced that HySp5 can mediate at least some effects of Wnt/β-catenin signaling during zebrafish gastrulation, a mediation that requires its DNA-binding activity.

Supplementary Tables

Supplementary Table 1. Cloning, siRNAs, qPCR and ChIP-qPCR primer sequences

(a) List of cloning primers

	Primer name	Sequence
**HySp5:Luc**	HySp5 promoter Forward	CTAGTTCTAATTTAGCTCTATTACGTTCGC
	HySp5 promoter Reverse	GAAACCGCCATCTTATCTTAAATAGCTTCGG
**ZfSp5a-ΔDBD**	ZfSp5a-ΔDBD Forward	CAGAACAAGAAGAGCAAAAGTCACG
	ZfSp5a-ΔDBD Reverse	CTGTTTCTTCTTTCCGGGCTCA
**HyWnt3-ΔRep:Luc**	HyWnt3-ΔRep:Luc Forward	CTCGAGATCTGCGATCTAAG
	HyWnt3-ΔRep:Luc Reverse	GCGGTTAGTTAAATCAAACC
**pGEM-T-Easy-HySp5**	HySp5 Forward (Sp5-For1)	AATTACTCACAAAAACTTT
	HySp5 Reverse (Sp5-Rev1)	TAAGGTGACTAGTTTTACC
**pGEM-T-Easy-HyWnt3**	HyWnt3 Forward	ATGGGCACGACGCGTTATAA
	HyWnt3 Reverse	CTATTTACAGGTGTATTCAG
**pGEM-T-Easy-HyBra1**	HyBra1 Forward	TGGAAGGCGAATGTTTCCTG
	HyBra1 Reverse	TTCGGTGATACGGTGATGGA

(b) List of siRNAs

HySp5 siRNA-1	UUA ACG AGC ACC ACA UAA A
HySp5 siRNA-2	CUA CAA CAU CCC ACA UAU A
HySp5 siRNA-3	GCA GCA CGU AUG UCA UAU U
β-catenin siRNA-1	UCA ACC UAA CAG ACA ACA A
β-catenin siRNA-2	UGA GGA GCU AUA CUU AUG A
β-catenin siRNA-3	ACG ACU CUC UGU UGA AUU A
scramble siRNA	AGGUAGUGUAAUCGCCUUG

(c) List of qPCR primers

HySp5 Forward	CCAGGGTGCGGAAAGGTT
HySp5 Reverse	CCAGCATGCCATCTTAAATGAG
Wnt3 Forward	GAGTTGACGGTTGCGAACTT
Wnt3 Reverse	ACATGAAACCTTGCAACACCA
β-catenin Forward	TACGCAATGTTGTTGGTGCT
β-catenin Reverse	GCTTCAATTCGATGGCCTAA
Bra1 Forward	ATAGATTGGTATCCGTGCGG
Bra1 Reverse	GGAAACTGAGGCGGATACCA
TBP Forward	AAGCGATTTGCAGCAGTTAT
TBP Reverse	GCTCTTCACTTTTTGCTCCA

(d) List of ChIP-qPCR primers

HySp5 promoter (*Hm-105*)	
PP1-F	TAAGCTGTCTCCATTTCAACCA
PP1-R	AATATTTGTTAAGTGTTTTTCGTTGG
PP2-F	TATCTTTTCCGCCTTACGTATTC
PP2-R	ACTGAGAAATGGCGCGTTG
PP3-F	CAGAGAAAATATGATCGCAACG
PP3-R	GAAACCGCCATCTTATCTTAAA
ZfWnt3 promoter	
PP1-F	TCTGAAGAGAAAGGGGCAAA
PP1-R	ACCCTCTCCTCACACACGTC
PP2-F	GCAAGCAACATGGGACAATA
PP2-R	ATGTAGGTTCCGGCCAATTT
PP3-F	ACAGCTGGGTTTCCTTGATG
PP3-R	AGGCTGGGAGGGAATAAGAA

Supplementary Table 2. DNA constructs used in this study

Name	Abbreviation	Reference	Source
pGL3-*HySp5*-2992	**HySp5:Luc**	/	This study
pGL3-*HyWnt3*-2149	**HyWnt3:Luc**	/	This study
pGL3-*HyWnt3*-1763	**HyWnt3-ΔRep:Luc**	/	This study
pGL3-*HyWnt3*-1858	**HyWnt3-ΔRep-D1:Luc**	/	This study
pGL3-*HyWnt3*-1864	**HyWnt3-ΔRep-D2:Luc**	/	This study
pGL3-*HyWnt3*-1959	**HyWnt3-ΔRep-D3:Luc**	/	This study
pGL3-*HyWnt3*-1953	**HyWnt3-ΔRep-D4:Luc**	/	This study
pEGFP-*ZfWnt3*-3997	**ZfWnt3-promoter**	Ref.[10]	Cathleen Teh (gift)
pGL3-*ZfWnt3*-3997	**ZfWnt3:Luc**	/	This study
pGL3	**no-prom:Luc**	Ref.[11]	Zbynek Kozmik (gift)
pCS2+		www.addgene.org/vector-database/2295/	
pCS2+-HySp5-FL	**HySp5-420**	/	This study
pCS2+-HySp5-ΔDBD	**HySp5-337**	/	This study
pCS2+-HySp5-ΔSP	**HySp5-310**	/	This study
pCS2+-HySp5-ΔSP-ΔDBD	**HySp5-227**	/	This study
pCS2+-ZfSp5a-FL	**ZfSp5a-337**	/	This study
pCS2+-ZfSp5a-ΔDBD	**ZfSp5a-289**	/	This study
pCS2+-ZfSp5l1-FL	**ZfSp5l1**	/	This study
ZE14 pCS2P+ wnt8 ORF1	**ZfWnt8**	Ref.[9]	Addgene # 17048
pcDNA-Wnt3	**huWnt3**	Ref.[12]	Addgene # 35909
pcDNA6-huLRP6-v5	**huLRP6**	Ref.[13]	Bart Willimans (gift)
pFLAG-CMV-hu-β-CateninΔ45	**huΔβ-cat**	Ref.[14]	Ariel Ruiz i Altaba (gift)
pCAG-FLAG-TCF-1	**TCF1**	Ref.[15]	Junichiro Yasunaga (gift)
pGEM-T-Easy-HyWnt3-1092	/	/	This study
pGEM-T-Easy-HyBra1-635	/	/	This study
pGEM-T-Easy-HySp5-557	/	/	This study
pGL4.74[hRluc/TK]		www.promega.com/-/media/files/resources/protocols/product-information-sheets/a/pgl474-vector.pdf?la=en	

Supplementary References

1 Schaeper, N. D., Prpic, N. M. & Wimmer, E. A. A clustered set of three Sp-family genes is ancestral in the Metazoa: evidence from sequence analysis, protein domain structure, developmental expression patterns and chromosomal location. *BMC Evol Biol* **10**, 88, doi:10.1186/1471-2148-10-88 (2010).

2 Wenger, Y., Buzgariu, W. & Galliot, B. Loss of neurogenesis in Hydra leads to compensatory regulation of neurogenic and neurotransmission genes in epithelial cells. *Philos Trans R Soc Lond B Biol Sci* **371**, 20150040, doi:10.1098/rstb.2015.0040 (2016).

3 Anton-Erxleben, F., Thomas, A., Wittlieb, J., Fraune, S. & Bosch, T. C. Plasticity of epithelial cell shape in response to upstream signals: a whole-organism study using transgenic Hydra. *Zoology (Jena)* **112**, 185-194, doi:10.1016/j.zool.2008.09.002
S0944-2006(09)00002-6 [pii] (2009).

4 Wittlieb, J., Khalturin, K., Lohmann, J. U., Anton-Erxleben, F. & Bosch, T. C. Transgenic Hydra allow in vivo tracking of individual stem cells during morphogenesis. *Proc Natl Acad Sci U S A* **103**, 6208-6211 (2006).

5 Hemmrich, G. *et al.* Molecular signatures of the three stem cell lineages in hydra and the emergence of stem cell function at the base of multicellularity. *Mol Biol Evol* **29**, 3267-3280, doi:10.1093/molbev/mss134 (2012).

6 Zhang, X., Peterson, K. A., Liu, X. S., McMahon, A. P. & Ohba, S. Gene regulatory networks mediating canonical Wnt signal-directed control of pluripotency and differentiation in embryo stem cells. *Stem Cells* **31**, 2667-2679, doi:10.1002/stem.1371 (2013).

7 Kennedy, M. W. *et al.* Sp5 and Sp8 recruit beta-catenin and Tcf1-Lef1 to select enhancers to activate Wnt target gene transcription. *Proc Natl Acad Sci U S A* **113**, 3545-3550, doi:10.1073/pnas.1519994113 (2016).

8 Pelegri, F. & Maischein, H. M. Function of zebrafish beta-catenin and TCF-3 in dorsoventral patterning. *Mech Dev* **77**, 63-74 (1998).

9 Lekven, A. C., Thorpe, C. J., Waxman, J. S. & Moon, R. T. Zebrafish wnt8 encodes two wnt8 proteins on a bicistronic transcript and is required for mesoderm and neurectoderm patterning. *Dev Cell* **1**, 103-114 (2001).

10 Teh, C., Sun, G., Shen, H., Korzh, V. & Wohland, T. Modulating the expression level of secreted Wnt3 influences cerebellum development in zebrafish transgenics. *Development* **142**, 3721-3733, doi:10.1242/dev.127589 (2015).

11 Fujimura, N. *et al.* Wnt-mediated down-regulation of Sp1 target genes by a transcriptional repressor Sp5. *J Biol Chem* **282**, 1225-1237, doi:10.1074/jbc.M605851200 (2007).

12 Najdi, R. *et al.* A uniform human Wnt expression library reveals a shared secretory pathway and unique signaling activities. *Differentiation* **84**, 203-213, doi:10.1016/j.diff.2012.06.004 (2012).

13 Holmen, S. L., Salic, A., Zylstra, C. R., Kirschner, M. W. & Williams, B. O. A novel set of Wnt-Frizzled fusion proteins identifies receptor components that activate beta -catenin-dependent signaling. *J Biol Chem* **277**, 34727-34735, doi:10.1074/jbc.M204989200 (2002).

14 Melotti, A. *et al.* The river blindness drug Ivermectin and related macrocyclic lactones inhibit WNT-TCF pathway responses in human cancer. *EMBO Mol Med* **6**, 1263-1278, doi:10.15252/emmm.201404084 (2014).

15 Ma, G., Yasunaga, J., Fan, J., Yanagawa, S. & Matsuoka, M. HTLV-1 bZIP factor dysregulates the Wnt pathways to support proliferation and migration of adult T-cell leukemia cells. *Oncogene* **32**, 4222-4230, doi:10.1038/onc.2012.450 (2013).
