# Supplementary material for: An evolutionarily-conserved Wnt3/β-catenin/Sp5 feedback loop restricts head organizer activity in Hydra
Source: Nat Commun. 2019 Jan 18;10:312. doi: 10.1038/s41467-018-08242-2 (PMC6338789; doi:10.1038/s41467-018-08242-2)
Supplement: Supplementary file 3 — Description of Additional Supplementary Files [file 41467_2018_8242_MOESM3_ESM.docx]

**Title:** Supplementary Movie 1.
**Description:** Feeding behavior of an intact scramble(RNAi) control animal

**Title:** Supplementary Movie 2.
**Description:** Feeding behavior of an intact HySp5(RNAi) animal Note that the ectopic heads that developed in HySp5(RNAi) animals normally catch preys, here Artemia.

**Title:** Supplementary Movie 3.
**Description:** Feeding behavior of a head-regenerating scramble(RNAi) control animal at 5 days post-amputation (dpa)

**Title:** Supplementary Movie 4.
**Description:** Feeding behavior of a head-regenerating HySp5(RNAi) animal at 5 days post amputation (dpa) Note that the ectopic heads that regenerated in HySp5(RNAi) animals normally catch preys, here Artemia.

**Title**: Supplementary Data 1

**Description**: Identification of Hydra head inhibitor candidates

**Title**: Supplementary Data 2

**Description**: ChIP-seq and RNA-seq data analysis

**Title**: Supplementary Data 3

**Description**: HySp5 and ZfSp5 direct transcriptional targets and associated gene ontology terms

**Title**: Supplementary Data 4

**Descriptio**n: Overexpression of Sp5 in zebrafish
