# Supplementary material for: An evolutionarily-conserved Wnt3/β-catenin/Sp5 feedback loop restricts head organizer activity in Hydra
Source: Nat Commun. 2019 Jan 18;10:312. doi: 10.1038/s41467-018-08242-2 (PMC6338789; doi:10.1038/s41467-018-08242-2)
Supplement: Supplementary file 12 — Reporting Summary [file 41467_2018_8242_MOESM12_ESM.pdf]

nature research

Corresponding author(s): Brigitte Galliot

Reporting Summary

Nature Research wishes to improve the reproducibility of the work that we publish. This form provides structure for consistency and transparency in reporting. For further information on Nature Research policies, see Authors & Referees and the Editorial Policy Checklist.

Statistical parameters

When statistical analyses are reported, confirm that the following items are present in the relevant location (e.g. figure legend, table legend, main text, or Methods section).

n/a	Confirmed	
☐	☒	The exact sample size (*n*) for each experimental group/condition, given as a discrete number and unit of measurement
☐	☒	An indication of whether measurements were taken from distinct samples or whether the same sample was measured repeatedly
☐	☒	The statistical test(s) used AND whether they are one- or two-sided<br>*Only common tests should be described solely by name; describe more complex techniques in the Methods section.*
☒	☐	A description of all covariates tested
☒	☐	A description of any assumptions or corrections, such as tests of normality and adjustment for multiple comparisons
☐	☒	A full description of the statistics including central tendency (e.g. means) or other basic estimates (e.g. regression coefficient) AND variation (e.g. standard deviation) or associated estimates of uncertainty (e.g. confidence intervals)
☒	☐	For null hypothesis testing, the test statistic (e.g. *F*, *t*, *r*) with confidence intervals, effect sizes, degrees of freedom and *P* value noted<br>*Give P values as exact values whenever suitable.*
☒	☐	For Bayesian analysis, information on the choice of priors and Markov chain Monte Carlo settings
☒	☐	For hierarchical and complex designs, identification of the appropriate level for tests and full reporting of outcomes
☒	☐	Estimates of effect sizes (e.g. Cohen's *d*, Pearson's *r*), indicating how they were calculated
☐	☒	Clearly defined error bars<br>*State explicitly what error bars represent (e.g. SD, SE, CI)*

Our web collection on statistics for biologists may be useful.

Software and code

Policy information about availability of computer code

Data collection	No software was used.
Data analysis	Prism 7 was used for all statistical analysis. Bowtie2 (version 2.2.6.2) was used for the mapping of ChIP-seq reads and significantly enriched regions were identified with MACS2 (version 2.1.0.20151222.0). MEME-ChIP suite was used to identify transcription factor binding sites. R (version 3.5.1) with Rstudio (Version 1.1.456) GenomicRanges (version 1.32.6) and the ChipEnrich (version 2.4.0) packages were used to process and analyse ChIPseq data.<br>STAR RNA-seq aligner was used to map RNAseq reads. DESeq2 was used to perform differential gene expression analysis. The R packages ade4 (version 1.7-13) and gplot (version 3.0.1) were used to analyze and plot RNAseq data.<br><br>PhyML 3.0 was used to calculate phylogentic trees.<br><br>Further details are in the Methods section.

For manuscripts utilizing custom algorithms or software that are central to the research but not yet described in published literature, software must be made available to editors/reviewers upon request. We strongly encourage code deposition in a community repository (e.g. GitHub). See the Nature Research guidelines for submitting code & software for further information.

Data

Policy information about availability of data

All manuscripts must include a data availability statement. This statement should provide the following information, where applicable:

- Accession codes, unique identifiers, or web links for publicly available datasets
- A list of figures that have associated raw data
- A description of any restrictions on data availability

The Hydra Sp5 sequence was deposited at GenBank (MG437301). The genome assemblies and reads are available under the BioProject PRJNA419866. ChIP-seq and RNA-seq experiments have been deposited with the GEO database under the following accessions: GSE121321.

Field-specific reporting

Please select the best fit for your research. If you are not sure, read the appropriate sections before making your selection.

☒ Life sciences ☐ Behavioural & social sciences ☐ Ecological, evolutionary & environmental sciences

For a reference copy of the document with all sections, see nature.com/authors/policies/ReportingSummary-flat.pdf

Life sciences study design

All studies must disclose on these points even when the disclosure is negative.

Sample size	The sample size was not predetermined by any statistical method.
Data exclusions	No samples were excluded from the analysis.
Replication	All experiments were repeated and the data presented in this study is based on at least two to three independent experiments. The number of repeats are given in the figure legends.
Randomization	The animals were selected randomly for all experiments.
Blinding	The investigators were not blinded during data collection and analysis.

Reporting for specific materials, systems and methods

Materials & experimental systems

n/a	Involved in the study
☐	☒ Unique biological materials
☐	☒ Antibodies
☐	☒ Eukaryotic cell lines
☒	☐ Palaeontology
☐	☒ Animals and other organisms
☒	☐ Human research participants

Methods

n/a	Involved in the study
☐	☒ ChIP-seq
☒	☐ Flow cytometry
☒	☐ MRI-based neuroimaging

Unique biological materials

Policy information about availability of materials

Obtaining unique materials	The pEGFP-Wnt3 plasmid is available upon request from Dr. Cathleen Teh. The pcDNA6-hLRP6-v5 is available upon request from Dr. Bart Williams. All other materials are commercially available.

Antibodies

Antibodies used	The following antibodies were used in this study: anti-DIG-AP (Roche, 11093274910), anti-RFamide (Gimmelikhuijzen and Graff, 1985), anti-rabbit Alexa488 (ThermoFisher Scientific, A21206), anti-HA (Novus Biologicals, NB600-363), normal rabbit IgG (Merck Millipore, 12-370), anti-beta-catenin (BD Biosciences, 610153), anti-TCF-1 (Santa Cruz Biotechnology, sc-271453), anti-rabbit HRP light chain (Abcam, ab99697), anti-mouse HRP heavy and light chain (Promega, W402B).

Validation	All reagents were optimized and validated by the companies.

Eukaryotic cell lines

Policy information about cell lines

Cell line source(s)	HEK293T cells were a gift from the laboratory of Ariel Ruiz i Altaba (University of Geneva, Medical School).
Authentication	No authentication was used as the cell line was directly obtained from the Altaba laboratory.
Mycoplasma contamination	HEK293T was not tested for mycoplasma contamination.
Commonly misidentified lines (See ICLAC register)	No commonly misidentified lines were used in this study.

Animals and other organisms

Policy information about studies involving animals; ARRIVE guidelines recommended for reporting animal research

Laboratory animals	Hydra vulgaris of the strain Jussy and AEP were used for all experiments
Wild animals	n/a
Field-collected samples	n/a

ChIP-seq

Data deposition

☒ Confirm that both raw and final processed data have been deposited in a public database such as GEO.

☒ Confirm that you have deposited or provided access to graph files (e.g. BED files) for the called peaks.

Data access links
May remain private before publication.

https://www.ncbi.nlm.nih.gov/geo/query/acc.cgi?acc=GSE121321

Files in database submission

ChIP_hySp5_dDBD_rep1.bigwig
ChIP_hySp5_dDBD_rep2.bigwig
ChIP_hySp5_rep1.bigwig
ChIP_hySp5_rep2.bigwig
ChIP_zfSp5_dDBD_rep1.bigwig
ChIP_zfSp5_dDBD_rep2.bigwig
ChIP_zfSp5_rep1.bigwig
ChIP_zfSp5_rep2.bigwig
ChIP_HySp5_dDBD merged_rep.bigwig
ChIP_HySp5 merged_rep.bigwig
ChIP_ZfSp5_dDBD merged_rep.bigwig
ChIP_ZfSp5 merged_rep.bigwig
ChIP_HySp5_dDBD_rep1.fastq.gz
ChIP_HySp5_dDBD_rep2.fastq.gz
ChIP_HySp5_rep1.fastq.gz
ChIP_HySp5_rep2.fastq.gz
ChIP_ZfSp5_dDBD_rep1.fastq.gz
ChIP_ZfSp5_dDBD_rep2.fastq.gz
ChIP_ZfSp5_rep1.fastq.gz
ChIP_ZfSp5_rep2.fastq.gz
TIC_HySp5_dDBD_rep1.fastq.gz
TIC_HySp5_dDBD_rep2.fastq.gz
TIC_HySp5_rep1.fastq.gz
TIC_HySp5_rep2.fastq.gz
TIC_ZfSp5_dDBD_rep1.fastq.gz
TIC_ZfSp5_dDBD_rep2.fastq.gz
TIC_ZfSp5_rep1.fastq.gz
TIC_ZfSp5_rep2.fastq.gz
HySp5_narrow_peaks.bed
ZfSp5_narrow_peaks.bed

Genome browser session
(e.g. UCSC)

https://genome.ucsc.edu/cgi-bin/hgTracks?hgS_doOtherUser=submit&hgS_otherUserName=leonardo.beccari&hgS_otherUserSessionName=sp5%20chip%2Dmapping%20galxyHg19

Methodology

Replicates	We performed two ChIPseq replicates per experimental condition. For ChIP-qPCR experiments testing the regulation of HySp5 over the HySp5 promoter and for the assay of ZfSp5 vs ZfWnt3 promoter, 2 independent biological replicates were performed. In each experiment 2 technical replicates per condition were used.
Sequencing depth	All ChIP-seq experiments were sequenced with 50bp read length. The number of total and uniquely mapped reads (versus the human genome -Hg19) per each sample are reported below:<br>TIC_HySp5_rep1: 51706980 unpaired reads, 22901696 (44.29%) were uniquely mapped reads<br>TIC_HySp5_dDBD_rep1: 58705475 unpaired reads, 27644869 (47.09%) were uniquely mapped reads<br>ChIP_ HySp5_rep1: 62429415 unpaired reads, 43481688 (69.65%) were uniquely mapped reads<br>ChIP_HySp5_dDBD_rep1: 57739936 unpaired reads, 40274642 (69.75%) were uniquely mapped reads<br>TIC_ZfSp5_rep1: 91054273 unpaired reads, 39272401 (43.13%) were uniquely mapped reads<br>TIC_ZfSp5_dDBD_rep1: 85464593 unpaired reads, 36110069 (42.25%) were uniquely mapped reads<br>ChIP_ ZfSp5_rep1: 105866336 unpaired reads, 73438712 (69.37%) were uniquely mapped reads<br>ChIP_ZfSp5_dDBD_rep1: 64959297 unpaired reads, 45549880 (70.12%) were uniquely mapped reads<br><br>TIC_HySp5_rep2: 23516719 unpaired reads, 8928171 (37.97%) were uniquely mapped reads<br>TIC_HySp5_dDBD_rep2: 24334935 unpaired reads, 9326749 (38.33%) were uniquely mapped reads<br>ChIP_ HySp5_rep2: 29834762 unpaired reads, 20734950 (69.50%) were uniquely mapped reads<br>ChIP_HySp5_dDBD_rep2: 28084438 unpaired reads, 19575313 (69.70%) were uniquely mapped reads<br>TIC_ZfSp5_rep2: 28503614 unpaired reads, 14989830 (52.59%) were uniquely mapped reads<br>TIC_ZfSp5_dDBD_rep2: 27204089 unpaired reads, 14314585 (52.62%) were uniquely mapped reads<br>ChIP_ ZfSp5_rep2: 28019185 unpaired reads, 19481192 (69.53%) were uniquely mapped reads<br>ChIP_ZfSp5_dDBD_rep2: 24804495 unpaired reads, 17370818 (70.03%) were uniquely mapped reads
Antibodies	anti-HA antibody (NB600-363, Novus Biologicals): 4 ug/sample
Peak calling parameters	Significantly enriched regions were identified using MACS2: (Zhang et al., 2008) (version 2.1.0.20151222.0).<br>Working parameters: gsize 2451960000 --bw=300 --ratio 1.0 --slocal 1000 --llocal 10000 --call-summits --keep-dup 1 --bdg --qvalue 0.05
Data quality	Total input chromatin and DNA obtained from a mutant version of Sp5, lacking the DNA binding domain, was used as negative control in each of our ChIP experiments showing no significant enrichment in the region bound by Hydra and zebrafish SP5 proteins.<br>For zfSp5: 70488 peaks with FDR>5% and fold enrichment >5<br>For HySp5: 29821 peaks with FDR>5% and fold enrichment >5
Software	Adapters and bad quality bases were removed with cutadapt (ref Martin et al 2011 version 1.8 options -m 15 -q 30 -a GATCGGAAGAGCACACGTCTGAACTCCAGTCAC for ChIP). Then reads were mapped using bowtie2 (ref Langmead et al. 2012 version 2.2.4 default parameters).<br>The peaks and the coverage were obtained as the output of MACS2 (ref Zhang et al 2008 version 2.1.1.20160309 command line: macs2 callpeak -t input.bam --call-summits -B). R and Rstudio softwares (Version 3.2.5 and Version 1.0.153, respectively) were used to normalize each sample by their respective millions of uniquely mapped reads.
